# Investigation of Cyber-Security and Cyber-Crimes in Oil and Gas Sectors Using the Innovative Structures of Complex Intuitionistic Fuzzy Relations

**DOI:** 10.3390/e23091112

**Published:** 2021-08-27

**Authors:** Naeem Jan, Abdul Nasir, Mohsin S. Alhilal, Sami Ullah Khan, Dragan Pamucar, Abdulrahman Alothaim

**Affiliations:** 1 Department of Mathematics, Institute of Numerical Sciences, Gomal University, Dera Ismail Khan 29050, Pakistan; naeem.jan.7300@gmail.com (N.J.); theabdulnasir@gmail.com (A.N.); gomal85@gmail.com (S.U.K.); 2 Department of Information Systems, College of Computer and Information Sciences, King Saud University, Riyadh 11543, Saudi Arabia; 3 Department of Logistics, Military Academy, University of Defence in Belgrade, 11 000 Belgrade, Serbia; dragan.pamucar@va.mod.gov.rs

**Keywords:** block chain, Cartesian product, complex intuitionistic fuzzy relation, complex intuitionistic fuzzy set, cyber-security, Hasse diagram, oil and gas industry

## Abstract

Recently, there has been enormous development due to advancements in technology. Industries and enterprises are moving towards a digital system, and the oil and gas industries are no exception. There are several threats and risks in digital systems, which are controlled through cyber-security. For the first time in the theory of fuzzy sets, this research analyzes the relationships between cyber-security and cyber-crimes in the oil and gas sectors. The novel concepts of complex intuitionistic fuzzy relations (CIFRs) are introduced. Moreover, the types of CIFRs are defined and their properties are discussed. In addition, an application is presented that uses the Hasse diagram to make a decision regarding the most suitable cyber-security techniques to implement in an industry. Furthermore, the omnipotence of the proposed methods is explained by a comparative study.

## 1. Introduction

The techniques and methods used for reasoning, modeling and computing are mostly of precise, deterministic and crisp nature. The term crisp refers to the concept of a dichotomy, i.e., yes or no rather than more or less. In conventional dual logic, a statement is either true or false—there are no other possibilities. In general, precision implies that the models are unambiguous and clear. Crisp knowledge can be modeled using the crisp/classical set theory, also known as Cantor’s set theory. Meanwhile, in mathematics, the uncertainty is modeled through the theory of fuzzy sets (FSs) and fuzzy logic (FL). In practice, uncertainty cannot be avoided. There have been numerous structures, techniques and formulations introduced to model uncertainty in the theory of FSs and FL. Each of these methods have their advantages, accompanied by some limitations, leaving some gaps. Thus, this paper focuses on the formulation of some novel structures and methods that aim to solve certain cyber-security and hacking issues faced by the oil and gas sectors.

The complex intuitionistic fuzzy set (CIFS) is a powerful tool in FS theory that is used to model ambiguity and uncertainty, but the concepts of relations have not yet been defined for CIFSs. This article introduces the concepts of relations in the theory of CIFSs. Using the Cartesian product of two CIFSs, the current study presents the definition of complex intuitionistic fuzzy relations (CIFRs). The formation of CIFSs is based on a pair of complex valued functions whose values and their sum are contained within the unit disc of a complex plane. These functions are called the membership grade and non-membership grade. The real portion of each of the complex valued functions is called the amplitude term and the imaginary portion is called the phase term. This structure enables the CIFSs and their relations to model multidimensional problems of uncertain nature. These CIFRs generalize fuzzy relations (FRs), complex fuzzy relations (CFRs) and intuitionistic fuzzy relations (IFRs). By setting the non-membership grade to zero, the CIFR is converted into CFR. Similarly, by setting the phase terms equal to zero, the outcome is the IFRs and FRs. However, the converse is not true. Hence, the CIFRs are superior to their predecessors because they can also handle problems in fuzzy, intuitionistic fuzzy, complex fuzzy and complex intuitionistic fuzzy environments. Besides CIFRs, this article also defines numerous types of CIFRs, such as the converse of a CIFR, complex intuitionistic equivalence fuzzy relation, complex intuitionistic pre-order fuzzy relation, complex intuitionistic partial order fuzzy relation, complex intuitionistic total order fuzzy relation, complex intuitionistic strict order fuzzy relation, composition of complex intuitionistic equivalence fuzzy relations and complex intuitionistic fuzzy equivalence classes. Moreover, some interesting properties of CIFRs have been discussed. In addition, a few useful results regarding CIFRs and their subtypes have also been presented. Furthermore, the Hasse diagram for the complex intuitionistic partial order fuzzy relations and sets is introduced. Additionally, some important definitions related to the Hasse diagram have been presented—for instance, maximum, minimum, maximal, minimal, supremum, infimum, upper and lower bound elements. Every definition in the article is supported by a suitable example.

Over time, businesses, enterprises and industries are becoming digitalized because digital systems render the organizational operations and processes smoother, more efficient and less time-consuming. Although digital systems have improved productivity and reduced expenditures, they also place businesses and industries at great risk of cybercrimes. The oil and gas industries are also being digitalized and the history shows that there have been many cyberattacks in these industries. Hackers steal sensitive data and destroy stored data and personal information about employees, such as credit card data. To overcome these risks, certain cyber-security measures, methods, software and techniques have been developed and implemented. This article analyzes the relationships among some cyber-securities and cyber-threats in the oil and gas industries using the proposed concepts of CIFRs. An application is also presented that uses the complex intuitionistic partial order fuzzy relations to select the best possible cyber-security measures among all the available options, as well as the most suitable security technique among the short-listed ones. Thus, the current study provides some very useful mathematical modeling techniques that can solve very complex problems. A number of security methods have been introduced by experts, and this paper focuses on selecting the best security measures for an organization. This will not only save time and money, but also simplify the maintenance of the security systems. Moreover, it is necessary to adopt a specific security system to protect against some particular cyber-threats. Finally, a comparison is carried out among the proposed method and the existing methods.

The rest of the paper is organized in the following way:

Section 2 discusses the literature review of the study.
Section 3 reviews some predefined concepts, which play the role of building blocks for this article.
Section 4 is focused on CIFRs and their properties. Moreover, some results are provided, and Hasse diagrams and the related definitions are presented.
Section 5 proposes a couple of applications of the CIFRs to study the relationships among cyber-security and cyber-risks in the oil and gas industries.
Section 6 compares the proposed structure with the existing competitors.
Section 7 concludes the article.

## 2. Literature Review

Klir [1] devised the crisp relations that are used to study the relationships among the crisp sets. However, the modeling of ambiguity/uncertainty has long been a challenge for researchers. In 1965, Zadeh [2] introduced the fuzzy sets (FSs) and logics, which are capable of modeling fuzziness, ambiguity and uncertainty. The elements of an FS are assigned a fuzzy function that takes values from the unit interval [0,1]. This fuzzy function is called the membership grade, and the number from a unit interval is called the fuzzy number (FN). In fuzzy set theory, the statements are of the more or less type. Mendel [3] introduced the concept of fuzzy relations (FRs) that are used to analyze the relationships among the FSs. These relations are also characterized by membership grades whose values range between 0 and 1. Torra [4] introduced the hesitant FSs; Zadeh [5] presented the FSs as a basis for possibility theory; Negoita and Ralescu [6] applied the FSs to system analysis; Goguen [7] defined the L-FSs; and Laengle et al. [8] proposed a bibliometric analysis of FSs. Using genetic algorithm based on FSs, Xu et al. [9] optimized many-objective flow shop scheduling and Mewada et al. [10] extrapolated a fuzzy system with applications.

Ramot et al. [11] considered altering the membership grade and proposed the idea of complex fuzzy sets (CFSs). The elements of CFSs are assigned a complex fuzzy function that attains values from the unit circle in a complex plane. Obviously, the complex fuzzy function is called the membership grade, but here, a membership grade is a complex number, in contrast to a real number in FSs. Therefore, a complex membership grade has two parts: the amplitude term and phase term. These sets can model the ambiguity with phase changes and time periods. Furthermore, Ramot et al. [12] defined the complex fuzzy relations (CFRs) for CFSs; Pedrycz [13] worked on FRs and relational computing; Yu et al. [14] provided the uncertainty measures for FRs with applications; De Baets and Kerre [15] proposed the applications of FRs. Based on FRs, Tamura et al. [16] presented the pattern classification; Bhattacharya and Mukherjee [17] offered the FRs and fuzzy groups; Braae and Rutherford [18] used FRs in a control setting. Zhang et al. [19] introduced the operation properties and δ-equalities of CFSs; Yazdanbakhsh and Dick [20] systematically reviewed the CFSs and logic; Tamir et al. [21] presented an overview of CFSs and CF logic along with their applications; Ma et al. [22] proposed a method for multiple periodic factor prediction problems through CFSs, and Nasir et al. [23] used interval-valued CFRs in medical diagnosis and studied the lifespan of patients.

Zadeh’s fuzzy set theory had a drawback in that it only discussed the membership grade, with only minor discussion of the non-membership grade, which is the complement of the membership grade, i.e., 1− membership grade. Therefore, Atanassov [24] defined the intuitionistic fuzzy sets (IFSs) that are characterized by a pair of functions called the membership and non-membership grades. Both of the grades attain values from the unit interval provided that their sum does not exceed 1. The numbers in the [0,1] interval that an IFS assigns to its grades are known as intuitionistic fuzzy numbers (IFNs). Burillo et al. [25] introduced the concepts of intuitionistic fuzzy relations (IFRs) for IFSs. These sets discuss the relationship in the environment of intuitionistic fuzzy set theory through the membership and non-membership grades. De et al. [26] applied IFSs in medical diagnosis; Szmidt and Kacprzyk [27] found the distances between IFSs; De et al. [28] defined some operations on IFSs; Gerstenkorn and Manko [29] gave the correlation of IFSs; Buyukozkan and Uzturk [30] used interval-valued IFQFD for designing a smart fridge; Rani and Garg [31] applied the CIFRs in individual and group decision-making problems; Bustince and Burillo [32] proposed structures on IFRs, and Deschrijver and Kerre [33] studied the composition of IFRs.

The involvement of complex numbers in IFSs was investigated by Alkouri et al. [34], who proposed the idea of complex intuitionistic fuzzy sets (CIFSs). A CIFS assigns each of its elements a pair of functions whose values are complex numbers from a unit circle provided that their sum also belongs to the unit circle in the complex plane. Similar to CFSs, the membership and non-membership grades of CIFSs also consist of amplitude terms and phase terms. Thus, they can model uncertain events with time periods and phase alterations. Yaqoob et al. [35] and Nasir et al. [36,37] studied the complex relations and applied them in cellular networks and economic relationships. Ngan et al. [38] represented CIFS by quaternion numbers and applied them in decision-making, while Kumar and Bajaj [39] worked on the distance measures and entropies in CIF soft sets.

The world is developing and every organization is being digitalized. This ensures that the operations of organizations are smooth, time-efficient, systematized and secure as compared to non-digital infrastructures. In recent years, hackers and other criminals have targeted the digital industries. There are certain threats and risks to the digital systems that need to be countered by implementing specific techniques. The oil and gas sectors are not an exception and have been targeted recently. Scientists and engineers have carried out research to make these industries secure. Lamba [40] described measures to protect the ‘cybersecurity and resiliency’ of the oil, energy and gas infrastructures of a nation; Line et al. [41] discussed the cyber-security challenges in smart grids; Lu et al. [42] and Lakhanpal and Samuel [43] reviewed the opportunities, applications, risks and challenges of implementing block-chain technology in oil and gas sectors. Based on factor state space, Yang et al. [44] presented a new cyber-security risk evaluation method; Stergiopoulos et al. [45] surveyed attack patterns and carried out incident assessment in the oil and gas industries to evaluate the cyber-attacks in the oil and gas sector; Wanasinghe et al. [46] carried out a systematic review of internet of things in the oil and gas sector, and Bjerga and Aven [47] used some new risk perspectives for adaptive risk management in the oil and gas sector.

## 3. Preliminaries

In this section, some fundamental concepts are reviewed, such as fuzzy sets (FSs), complex FSs (CFSs), intuitionistic FSs (CFSs), complex IFSs (CIFSs), Cartesian product (CP) of two CFSs and complex fuzzy relations (CFRs). In addition, the definitions are supported by examples.

**Definition** **1** ([2])**.** *An FS* Ḯ *is characterized by a function* m:Ḯ→[0,1] *that assigns to each* u∈Ḯ *a fuzzy number (FN)* m(u)∈[0,1]. *The function* m *is called the membership grade. Henceforth, an FS* Ḯ *on a universal set* Ȿ *is of the following form*:

Ḯ={(u,m(u)):u∈Ȿ} 

**Example** **1.** Ḯ={(u,0.359),(v,0.654),(w,0.982),(x,0.234),(y,0.000),(z,1.000)} *is an FS*.

**Definition** **2** ([11])**.** *A CFS* Ḯ *is characterized by a function* $m_{C}:Ḯ\to Z$, *where* Z ϵ ℂ∋|Z|≤1. $m_{C}$ *assigns to each* u∈Ḯ *a complex number such that* $\left|m_{C}\left(u\right)\right|\in \left[0,1\right]$. *The function* $m_{C}\left(u\right)$ *is called the membership grade, defined as* $m_{C}\left(u\right)=\alpha \left(u\right)e^{\rho \left(u\right)2\pi i}$, *where* $i=\sqrt{-1}$ *,* α(u)∈[0,1] *is named the amplitude term and*  ρ(u)∈[0,1] *is named the phase term. Henceforth, a CFS* Ḯ *on a universal set* Ȿ *is of the following form*:

$$Ḯ=\left\{\left(u,\alpha \left(u\right)e^{\rho \left(u\right)2\pi i}\right):u\in Ȿ\right\}\;$$

**Example** **2.** $Ḯ=\left\{\begin{array}{c} \left(u,0.336e^{\left(0.526\right)2\pi i}\right),\left(v,0.619e^{\left(0.736\right)2\pi i}\right),\left(w,0.975e^{\left(0.128\right)2\pi i}\right),\\ \left(x,0.254e^{\left(0.672\right)2\pi i}\right),\left(y,0.000e^{\left(1.000\right)2\pi i}\right),\left(z,1.000e^{\left(0.000\right)2\pi i}\right) \end{array}\right\}$*is a CFS*.

**Definition** **3** ([12])**.** *The CP of CFSs* $Ḯ=\left\{\left(u,\alpha^{Ḯ}\left(u\right)e^{\rho^{Ḯ}\left(u\right)2\pi i}\right):u\in Ȿ\right\}$ *and*

$ʝ=\left\{\left(v,\alpha^{ʝ}\left(v\right)e^{\rho^{ʝ}\left(v\right)2\pi i}\right):v\in Ȿ\right\}$* is given by*

$$Ḯ\times ʝ=\left\{\left(\left(u,v\right),\alpha^{Ḯ\times ʝ}\left(u,v\right)e^{\rho^{Ḯ\times ʝ}\left(u,v\right)2\pi i}\right):u\in Ḯ,v\in ʝ\right\}$$

*where*$\alpha^{Ḯ\times ʝ}\left(u,v\right)=\min\left\{\alpha^{Ḯ}\left(u\right),\alpha^{ʝ}\left(v\right)\right\}$*and*$\rho^{Ḯ\times ʝ}\left(u,v\right)=\min\left\{\rho^{Ḯ}\left(u\right),\rho^{ʝ}\left(v\right)\right\}$.

**Definition** **4** ([12])**.** *Any subset of the CP of two CFSs is known as a complex fuzzy relation (CFR), which is symbolized by* R.

**Example** **3.** *Let*$Ḯ=\left\{\begin{array}{c} \left(u,0.3e^{\left(0.5\right)2\pi i}\right),\left(v,0.6e^{\left(0.7\right)2\pi i}\right),\left(w,0.9e^{\left(0.1\right)2\pi i}\right) \end{array}\right\}$*and*$ʝ=\left\{\left(x,0.2e^{\left(0.6\right)2\pi i}\right),\left(y,0e^{\left(1\right)2\pi i}\right),\left(z,1e^{\left(0\right)2\pi i}\right)\right\}$, *then the CP is found to be*

$$Ḯ\times ʝ=\left\{\begin{array}{c} \begin{array}{c} \left(\left(u,x\right),0.2e^{\left(0.5\right)2\pi i}\right),\left(\left(u,y\right),0e^{\left(0.5\right)2\pi i}\right),\left(\left(u,z\right),0.3e^{\left(0\right)2\pi i}\right) \end{array},\\ \begin{array}{c} \left(\left(v,x\right),0.2e^{\left(0.6\right)2\pi i}\right),\left(\left(v,y\right),0e^{\left(0.7\right)2\pi i}\right),\left(\left(v,z\right),0.6e^{\left(0\right)2\pi i}\right), \end{array}\\ \begin{array}{c} \left(\left(w,x\right),0.2e^{\left(0.1\right)2\pi i}\right),\left(\left(w,y\right),0e^{\left(0.1\right)2\pi i}\right),\left(\left(w,z\right),0.9e^{\left(0\right)2\pi i}\right) \end{array} \end{array}\right\}\;$$

**Figure 1:**
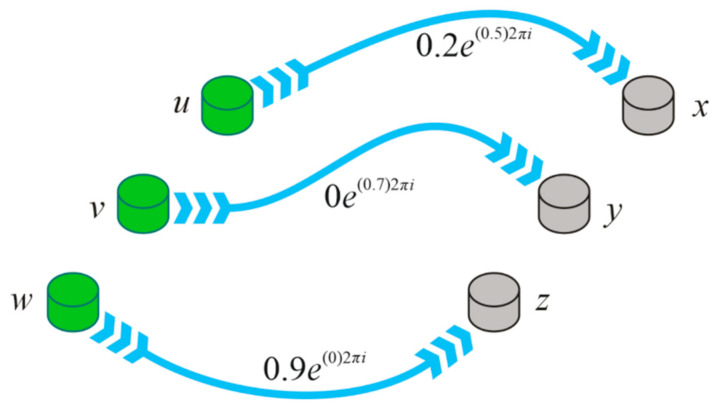
Complex fuzzy relation.

*The CFR*R* is given as follows (Figure 1*)

$$R=\left\{\left(\left(u,x\right),0.2e^{\left(0.5\right)2\pi i}\right),\left(\left(v,y\right),0e^{\left(0.7\right)2\pi i}\right),\left(\left(w,z\right),0.9e^{\left(0\right)2\pi i}\right)\right\}\;$$

**Definition** **5** ([24])**.** *An IFS* Ḯ *is characterized by a pair of functions* m,n:Ḯ→[0,1] *that are each assigned* u∈Ḯ *a pair of fuzzy numbers (FN)* m(u),n(u)∈[0,1], *provided that the sum* m(u)+n(u)≤1. *The function* m *is called the membership grade and the function* n *is called the non-membership grade. Henceforth, an IFS* Ḯ *on a universal set* Ȿ *is of the following form*:

Ḯ={(u,m(u),n(u)):u∈Ȿ}

**Example** **4.** Ḯ={(u,0.3,0.5),(v,0.6,0.2),(w,0.9,0.1)}*is an IFS*.

**Definition** **6** ([34])**.** *A CIFS* Ḯ *is characterized by a pair of complex valued functions* $m_{C},n_{C}:Ḯ\to Z$, *where* Z ϵ ℂ∋|Z|≤1. $m_{C}$ *and* $\;n_{C}$ *assigns to each* u∈Ḯ *a pair of complex numbers such that* $\left|m_{C}\left(u\right)\right|,\left|n_{C}\left(u\right)\right|\in \left[0,1\right]$ *and* $\left|m_{C}\left(u\right)\right|+\left|n_{C}\left(u\right)\right|\leq 1$. *The function* $m_{C}\left(u\right)$ *is called the membership grade and the function* $n_{C}\left(u\right)$ *is called the non-membership grade, which are defined as* $m_{C}\left(u\right)=\alpha_{m}\left(u\right)e^{\rho_{m}\left(u\right)2\pi i}$ *and* $n_{C}\left(u\right)=\alpha_{n}\left(u\right)e^{\rho_{n}\left(u\right)2\pi i}$, *where* $i=\sqrt{-1}$, $\alpha_{m}\left(u\right),\alpha_{n}\left(u\right)\in \left[0,1\right]$ *are called the amplitude terms of the membership and non-membership grades, respectively, and* $\;\rho_{m}\left(u\right),\rho_{n}\left(u\right)\in \left[0,1\right]$ *are called the phase terms of the membership and non-membership grades, respectively. Henceforth, a CIFS* Ḯ *on a universal set* Ȿ *is of the following form*:

$$Ḯ=\left\{\left(u,\alpha_{m}\left(u\right)e^{\rho_{m}\left(u\right)2\pi i},\alpha_{n}\left(u\right)e^{\rho_{n}\left(u\right)2\pi i}\right):u\in Ȿ\right\}\;$$

*provided that* $\alpha_{m}\left(u\right)+\alpha_{n}\left(u\right)\leq 1$ *and* $\rho_{m}\left(u\right)+\rho_{n}\left(u\right)\leq 1$.

**Example** **5.** $Ḯ=\left\{\begin{array}{c} \left(u,0.3e^{\left(0.5\right)2\pi i},0.3e^{\left(0.5\right)2\pi i}\right),\left(v,0.5e^{\left(0.7\right)2\pi i},0.4e^{\left(0.2\right)2\pi i}\right),\\ \left(w,0.1e^{\left(0.4\right)2\pi i},0.6e^{\left(0.6\right)2\pi i}\right) \end{array}\right\}$ *is a CIFS*.

## 4. Complex Intuitionistic Fuzzy Relations and Their Properties

This section introduces the novel concepts of CP of two CIFSs, complex intuitionistic fuzzy relations (CIFRs) and their types. Every definition is supported by a suitable example. Moreover, some interesting results for CIFRs are provided. Additionally, the Hasse diagram for the complex intuitionistic partial order relations is presented. The notions of maximum, minimum, maximal, minimal, supremum, infimum, upper and lower bounds are defined as well.

**Definition** **7.** *The CP of CIFSs*$Ḯ=\left\{\left(u,\alpha_{m}^{Ḯ}\left(u\right)e^{\rho_{m}^{Ḯ}\left(u\right)2\pi i},\alpha_{n}^{Ḯ}\left(u\right)e^{\rho_{n}^{Ḯ}\left(u\right)2\pi i}\right):u\in Ȿ\right\}$*and*$ʝ=\left\{\left(u,\alpha_{m}^{ʝ}\left(v\right)e^{\rho_{m}^{ʝ}\left(v\right)2\pi i},\alpha_{n}^{ʝ}\left(v\right)e^{\rho_{n}^{ʝ}\left(v\right)2\pi i}\right):v\in Ȿ\right\}$*is denoted and defined as*

$$Ḯ\times ʝ=\left\{\left(\left(u,v\right),\alpha_{m}^{Ḯ\times ʝ}\left(u,v\right)e^{\rho_{m}^{Ḯ\times ʝ}\left(u,u\right)2\pi i},\alpha_{n}^{Ḯ\times ʝ}\left(u,v\right)e^{\rho_{n}^{Ḯ\times ʝ}\left(u,v\right)2\pi i}\right):u\in Ḯ,v\in ʝ\right\}\;$$

*where*$\alpha_{m}^{Ḯ\times ʝ}\left(u,v\right)=\min\left\{\alpha_{m}^{Ḯ}\left(u\right),\alpha_{m}^{ʝ}\left(v\right)\right\}$, $\rho_{m}^{Ḯ\times ʝ}\left(u,v\right)=\min\left\{\rho_{m}^{Ḯ}\left(u\right),\rho_{m}^{ʝ}\left(v\right)\right\}$$\alpha_{n}^{Ḯ\times ʝ}\left(u,v\right)=\max\left\{\alpha_{n}^{Ḯ}\left(u\right),\alpha_{n}^{ʝ}\left(v\right)\right\}$*and*$\rho_{n}^{Ḯ\times ʝ}\left(u,v\right)=\max\left\{\rho_{n}^{Ḯ}\left(u\right),\rho_{n}^{ʝ}\left(v\right)\right\}$.

**Definition** **8.** *Any subset of the CP*Ḯ×ʝ*of two CIFSs*Ḯ*and*ʝ*is known as a complex intuitionistic fuzzy relation (CIFR) between*Ḯ*and*ʝ.

*Any subset of the CP*Ḯ×ʝ*is known as a CIFR on*Ḯ.

*A CIFR is symbolized by*R.

**Example** **6.** *Let*$Ḯ=\left\{\begin{array}{c} \left(u,0.3e^{\left(0.5\right)2\pi i},0.5e^{\left(0.4\right)2\pi i}\right),\left(v,0.6e^{\left(0.7\right)2\pi i},0.4e^{\left(0.3\right)2\pi i}\right) \end{array}\right\}$*and*$ʝ=\left\{\left(x,0.2e^{\left(0.2\right)2\pi i}0.6e^{\left(0.5\right)2\pi i}\right),\left(y,0e^{\left(1\right)2\pi i},1e^{\left(0\right)2\pi i}\right)\right\}$, *then the CP is found to be*

$$Ḯ\times ʝ=\left\{\begin{array}{c} \begin{array}{c} \left(\left(u,x\right),0.2e^{\left(0.2\right)2\pi i},0.6e^{\left(0.5\right)2\pi i}\right),\left(\left(u,y\right),0e^{\left(0.5\right)2\pi i},1e^{\left(0.4\right)2\pi i}\right) \end{array},\\ \begin{array}{c} \left(\left(v,x\right),0.2e^{\left(0.2\right)2\pi i},0.6e^{\left(0.5\right)2\pi i}\right),\left(\left(v,y\right),0e^{\left(0.7\right)2\pi i},1e^{\left(0.3\right)2\pi i}\right) \end{array} \end{array}\right\}$$

**Figure 2:**
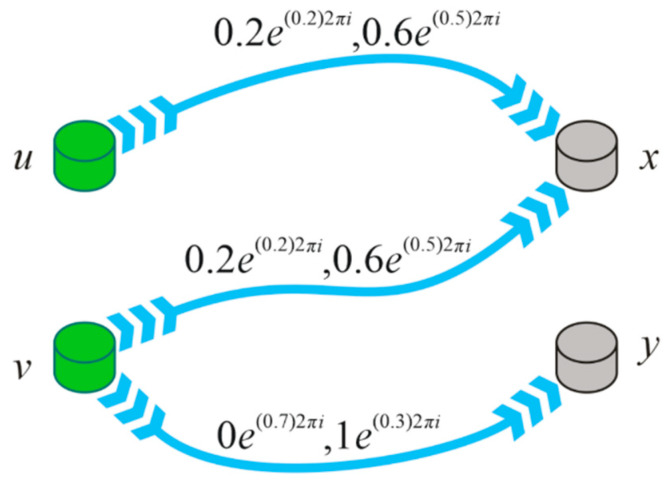
Complex intuitionistic fuzzy relation.

*The CIFR*R*between*Ḯ*and*ʝ* is given as follows (Figure 2)*

$$R=\left\{\begin{array}{c} \left(\left(u,x\right),0.2e^{\left(0.2\right)2\pi i},0.6e^{\left(0.5\right)2\pi i}\right),\left(\left(v,x\right),0.2e^{\left(0.2\right)2\pi i},0.6e^{\left(0.5\right)2\pi i}\right),\\ \left(\left(v,y\right),0e^{\left(0.7\right)2\pi i},1e^{\left(0.3\right)2\pi i}\right) \end{array}\right\}$$

**NOTE:** For convenience, (u,v) will be used to denote $\left(\left(u,v\right),\alpha_{m}^{Ḯ\times Ḯ}\left(u,v\right)e^{\rho_{m}^{Ḯ\times Ḯ}\left(u,v\right)2\pi i},\alpha_{n}^{Ḯ\times Ḯ}\left(u,v\right)e^{\rho_{n}^{Ḯ\times Ḯ}\left(u,v\right)2\pi i}\right)$ throughout this paper, unless otherwise stated.

**Definition** **9.** *Let*R*be a CIFR on a CIFS*Ḯ. *Then*,

1. R*is complex intuitionistic reflexive FR if*(u,u)∈R, ∀u∈Ḯ.
2. R*is complex intuitionistic symmetric FR if*∀u,v∈Ḯ.
  (u,v)∈R⇒(v,u)∈R.
3. R*is complex intuitionistic transitive FR if*∀u,v,w∈Ḯ.
  (u,v)∈R and (v,w)∈R⇒(u,w)∈R.
4. *A complex intuitionistic equivalence FR*R*on*Ḯ*possesses the following properties*:
  - *Complex intuitionistic reflexive*;
  - *Complex intuitionistic symmetric*;
  - *Complex intuitionistic transitive*.
5. *A complex intuitionistic preorder FR*R*on*Ḯ*possesses the following properties*:
  - *Complex intuitionistic reflexive*;
  - *Complex intuitionistic transitive*.
6. R*is complex intuitionistic antisymmetric FR if*∀u,v∈Ḯ.
  (u,v)∈R and (v,u)∈R⇒u=v.
7. *A complex intuitionistic partial order FR*R*on*Ḯ*possesses the following properties*:
  - *Complex intuitionistic reflexive*;
  - *Complex intuitionistic antisymmetric*;
  - *Complex intuitionistic transitive*.
  R* is also called the complex intuitionistic order FR*.
8. R*is complex intuitionistic complete FR if*∀u,v∈Ḯ.
  (u,v)∈R or (v,u)∈R.
9. *A complex intuitionistic linear order FR*R*on*Ḯ*possesses the following properties*:
  - *Complex intuitionistic reflexive*;
  - *Complex intuitionistic antisymmetric*;
  - *Complex intuitionistic transitive*;
  - *Complex intuitionistic complete*.
  *It is also called the complex intuitionistic total order FR*.
10. R*is complex intuitionistic irreflexive FR if*(u,u)∉R, ∀u∈Ḯ.
11. *A complex intuitionistic strict order FR*R*on*Ḯ*possesses the following properties*:
  - *Complex intuitionistic irreflexive*;
  - *Complex intuitionistic transitive*.

**Example** **7.** *For a CIFS*$Ḯ=\left\{\begin{array}{c} \left(x,0.20e^{\left(0.40\right)2\pi i}0.60e^{\left(0.50\right)2\pi i}\right),\\ \left(y,0.10e^{\left(0.40\right)2\pi i},0.10e^{\left(0.30\right)2\pi i}\right),\\ \left(z,0.60e^{\left(0.10\right)2\pi i},0.30e^{\left(0.80\right)2\pi i}\right) \end{array}\right\}$, *the CP*Ḯ×Ḯ*is found*

$$Ḯ\times Ḯ=\left\{\begin{array}{c} \left(\left(x,x\right),0.20e^{\left(0.40\right)2\pi i}0.60e^{\left(0.50\right)2\pi i}\right),\left(\left(x,y\right),0.10e^{\left(0.40\right)2\pi i}0.60e^{\left(0.50\right)2\pi i}\right),\\ \left(\left(x,z\right),0.20e^{\left(0.10\right)2\pi i}0.60e^{\left(0.80\right)2\pi i}\right),\left(\left(y,x\right),0.10e^{\left(0.40\right)2\pi i}0.60e^{\left(0.50\right)2\pi i}\right),\\ \left(\left(y,y\right),0.10e^{\left(0.40\right)2\pi i},0.10e^{\left(0.30\right)2\pi i}\right),\left(\left(y,z\right),0.10e^{\left(0.10\right)2\pi i}0.30e^{\left(0.80\right)2\pi i}\right),\\ \left(\left(z,x\right),0.20e^{\left(0.10\right)2\pi i}0.60e^{\left(0.80\right)2\pi i}\right),\left(\left(z,y\right),0.10e^{\left(0.10\right)2\pi i}0.30e^{\left(0.80\right)2\pi i}\right),\\ \left(\left(z,z\right),0.60e^{\left(0.10\right)2\pi i},0.30e^{\left(0.80\right)2\pi i}\right) \end{array}\right\}$$

*Then*,

1. *The complex intuitionistic equivalence fuzzy relation*$R_{1}$*on*Ḯ×Ḯ*is*
  $$R_{1}=\left\{\begin{array}{c} \left(\left(x,x\right),0.20e^{\left(0.40\right)2\pi i}0.60e^{\left(0.50\right)2\pi i}\right),\left(\left(x,y\right),0.10e^{\left(0.40\right)2\pi i}0.60e^{\left(0.50\right)2\pi i}\right),\\ \left(\left(y,x\right),0.10e^{\left(0.40\right)2\pi i}0.60e^{\left(0.50\right)2\pi i}\right),\left(\left(y,y\right),0.10e^{\left(0.40\right)2\pi i},0.10e^{\left(0.30\right)2\pi i}\right),\\ \left(\left(z,z\right),0.60e^{\left(0.10\right)2\pi i},0.30e^{\left(0.80\right)2\pi i}\right) \end{array}\right\}\;$$
2. *The complex intuitionistic partial order fuzzy relation*$R_{2}$*on*Ḯ×Ḯ*is*
  $$R_{2}=\left\{\begin{array}{c} \left(\left(x,x\right),0.20e^{\left(0.40\right)2\pi i}0.60e^{\left(0.50\right)2\pi i}\right),\left(\left(x,y\right),0.10e^{\left(0.40\right)2\pi i}0.60e^{\left(0.50\right)2\pi i}\right),\\ \left(\left(y,y\right),0.10e^{\left(0.40\right)2\pi i},0.10e^{\left(0.30\right)2\pi i}\right),\left(\left(z,y\right),0.10e^{\left(0.10\right)2\pi i}0.30e^{\left(0.80\right)2\pi i}\right),\\ \left(\left(z,z\right),0.60e^{\left(0.10\right)2\pi i},0.30e^{\left(0.80\right)2\pi i}\right) \end{array}\right\}\;$$
3. *The complex intuitionistic linear order fuzzy relation*$R_{3}$*on*Ḯ×Ḯ*is*
  $$R_{3}=\left\{\begin{array}{c} \left(\left(x,x\right),0.20e^{\left(0.40\right)2\pi i}0.60e^{\left(0.50\right)2\pi i}\right),\left(\left(x,y\right),0.10e^{\left(0.40\right)2\pi i}0.60e^{\left(0.50\right)2\pi i}\right),\\ \left(\left(x,z\right),0.20e^{\left(0.10\right)2\pi i}0.60e^{\left(0.80\right)2\pi i}\right),\left(\left(y,y\right),0.10e^{\left(0.40\right)2\pi i},0.10e^{\left(0.30\right)2\pi i}\right),\\ \left(\left(y,z\right),0.10e^{\left(0.10\right)2\pi i}0.30e^{\left(0.80\right)2\pi i}\right),\left(\left(z,z\right),0.60e^{\left(0.10\right)2\pi i},0.30e^{\left(0.80\right)2\pi i}\right) \end{array}\right\}\;$$
4. *The complex intuitionistic strict order fuzzy relation*$R_{4}$*on*Ḯ×Ḯ*is*
  $$R_{4}=\left\{\begin{array}{c} \left(\left(x,y\right),0.10e^{\left(0.40\right)2\pi i}0.60e^{\left(0.50\right)2\pi i}\right),\left(\left(x,z\right),0.20e^{\left(0.10\right)2\pi i}0.60e^{\left(0.80\right)2\pi i}\right),\\ \left(\left(y,z\right),0.10e^{\left(0.10\right)2\pi i}0.30e^{\left(0.80\right)2\pi i}\right) \end{array}\right\}\;$$

**Definition** **10.** *The converse of a CIFR* R *is defined as*

$$R^{c}=\left\{\left(v,u\right):\left(u,v\right)\in R\right\}\;$$

**Example** **8.** *For CIFSs*

$Ḯ=\left\{\left(w,0.4e^{\left(0.4\right)2\pi i}0.5e^{\left(0.5\right)2\pi i}\right),\left(x,0.2e^{\left(0.4\right)2\pi i}0.6e^{\left(0.5\right)2\pi i}\right)\right\}$ *and*

$ʝ=\left\{\left(y,0.1e^{\left(0.4\right)2\pi i},0.1e^{\left(0.3\right)2\pi i}\right),\left(z,0.6e^{\left(0.1\right)2\pi i},0.3e^{\left(0.8\right)2\pi i}\right)\right\}$, *the CP*Ḯ×ʝ*is found*

$$R=Ḯ\times ʝ=\left\{\begin{array}{c} \left(\left(w,y\right),0.1e^{\left(0.4\right)2\pi i}0.5e^{\left(0.5\right)2\pi i}\right),\left(\left(w,z\right),0.4e^{\left(0.1\right)2\pi i}0.5e^{\left(0.8\right)2\pi i}\right),\\ \left(\left(x,y\right),0.1e^{\left(0.4\right)2\pi i}0.6e^{\left(0.5\right)2\pi i}\right),\left(\left(x,z\right),0.2e^{\left(0.1\right)2\pi i}0.6e^{\left(0.8\right)2\pi i}\right) \end{array}\right\}\;$$

*Then, the converse of a CIFR* R *is given as*

$$R^{c}=\left\{\begin{array}{c} \left(\left(y,w\right),0.1e^{\left(0.4\right)2\pi i}0.5e^{\left(0.5\right)2\pi i}\right),\left(\left(y,x\right),0.1e^{\left(0.4\right)2\pi i}0.6e^{\left(0.5\right)2\pi i}\right),\\ \left(\left(z,w\right),0.4e^{\left(0.1\right)2\pi i}0.5e^{\left(0.8\right)2\pi i}\right),,\left(\left(z,x\right),0.2e^{\left(0.1\right)2\pi i}0.6e^{\left(0.8\right)2\pi i}\right) \end{array}\right\}\;$$

The complex intuitionistic equivalence fuzzy relations give rise to the concept of complex intuitionistic fuzzy equivalence classes, which are defined as follows.

**Definition** **11.** *Let*R*be a complex intuitionistic equivalence fuzzy relation on a CIFS*Ḯ. *For*u∈Ḯ, *a complex intuitionistic fuzzy equivalence class of*u*mod*R*is defined and symbolized as*

R[u]={v|(v,u)∈R}

**Example** **9.** *For a CIFS*$Ḯ=\left\{\begin{array}{c} \left(x,0.3e^{\left(0.4\right)2\pi i}0.5e^{\left(0.5\right)2\pi i}\right),\left(y,0.4e^{\left(0.3\right)2\pi i},0.2e^{\left(0.3\right)2\pi i}\right),\\ \left(z,0.8e^{\left(0.4\right)2\pi i},0.1e^{\left(0.6\right)2\pi i}\right) \end{array}\right\}$, *the CP*Ḯ×Ḯ*is found*

$$Ḯ\times Ḯ=\left\{\begin{array}{c} \left(\left(x,x\right),0.3e^{\left(0.4\right)2\pi i}0.5e^{\left(0.5\right)2\pi i}\right),\left(\left(x,y\right),0.3e^{\left(0.4\right)2\pi i}0.5e^{\left(0.5\right)2\pi i}\right),\\ \left(\left(x,z\right),0.3e^{\left(0.4\right)2\pi i}0.5e^{\left(0.6\right)2\pi i}\right),\left(\left(y,x\right),0.3e^{\left(0.4\right)2\pi i}0.5e^{\left(0.5\right)2\pi i}\right),\\ \left(\left(y,y\right),0.4e^{\left(0.3\right)2\pi i},0.2e^{\left(0.3\right)2\pi i}\right),\left(\left(y,z\right),0.4e^{\left(0.3\right)2\pi i}0.2e^{\left(0.6\right)2\pi i}\right),\\ \left(\left(z,x\right),0.3e^{\left(0.4\right)2\pi i}0.5e^{\left(0.6\right)2\pi i}\right),\left(\left(z,y\right),0.4e^{\left(0.3\right)2\pi i}0.2e^{\left(0.6\right)2\pi i}\right),\\ \left(\left(z,z\right),0.8e^{\left(0.4\right)2\pi i},0.1e^{\left(0.6\right)2\pi i}\right) \end{array}\right\}$$

*The complex intuitionistic equivalence fuzzy relation on* Ḯ *is*

$$R=\left\{\begin{array}{c} \left(\left(x,x\right),0.3e^{\left(0.4\right)2\pi i}0.5e^{\left(0.5\right)2\pi i}\right),\left(\left(x,y\right),0.3e^{\left(0.4\right)2\pi i}0.5e^{\left(0.5\right)2\pi i}\right),\\ \left(\left(y,x\right),0.3e^{\left(0.4\right)2\pi i}0.5e^{\left(0.5\right)2\pi i}\right),\left(\left(y,y\right),0.4e^{\left(0.3\right)2\pi i},0.2e^{\left(0.3\right)2\pi i}\right),\\ \left(\left(z,z\right),0.8e^{\left(0.4\right)2\pi i},0.1e^{\left(0.6\right)2\pi i}\right) \end{array}\right\}$$

*Then, the complex intuitionistic fuzzy equivalence class of*

1.
x *mod* R *is*
$$R\left[x\right]=\left\{\left(x,0.3e^{\left(0.4\right)2\pi i}0.5e^{\left(0.5\right)2\pi i}\right),\left(y,0.4e^{\left(0.3\right)2\pi i},0.2e^{\left(0.3\right)2\pi i}\right)\right\}\;$$
2.
y *mod* R *is*
$$R\left[y\right]=\left\{\left(x,0.3e^{\left(0.4\right)2\pi i}0.5e^{\left(0.5\right)2\pi i}\right),\left(y,0.4e^{\left(0.3\right)2\pi i},0.2e^{\left(0.3\right)2\pi i}\right)\right\}\;$$
3.
z *mod* R *is*
$$R\left[z\right]=\left\{\left(z,0.8e^{\left(0.4\right)2\pi i},0.1e^{\left(0.6\right)2\pi i}\right)\right\}$$

**Definition** **12.** *Let*R*be a CIFR on a CIFS*Ḯ. *Then, the complex intuitionistic composite FR*R∘R*is defined as*

*For any*(u,v)∈R*and*(v,w)∈R⇒(u,w)∈R∘R, ∀u,v,w∈U.

**Example** **10.** *Let*$R_{1}$*and*$R_{2}$*be two CIFRs on some CIFS*$Ḯ=\left\{\begin{array}{c} \left(x,0.3e^{\left(0.4\right)2\pi i}0.5e^{\left(0.5\right)2\pi i}\right),\left(y,0.4e^{\left(0.3\right)2\pi i},0.2e^{\left(0.3\right)2\pi i}\right),\left(z,0.8e^{\left(0.4\right)2\pi i},0.1e^{\left(0.6\right)2\pi i}\right) \end{array}\right\}$,

$$R_{1}=\left\{\begin{array}{c} \left(\left(x,y\right),0.3e^{\left(0.4\right)2\pi i}0.5e^{\left(0.5\right)2\pi i}\right),\left(\left(y,y\right),0.4e^{\left(0.3\right)2\pi i},0.2e^{\left(0.3\right)2\pi i}\right)\\ \left(\left(z,z\right),0.8e^{\left(0.4\right)2\pi i},0.1e^{\left(0.6\right)2\pi i}\right) \end{array}\right\}$$

$$R_{2}=\left\{\begin{array}{c} \left(\left(x,z\right),0.3e^{\left(0.4\right)2\pi i}0.5e^{\left(0.6\right)2\pi i}\right),\left(\left(y,x\right),0.3e^{\left(0.4\right)2\pi i}0.5e^{\left(0.5\right)2\pi i}\right),\\ \left(\left(z,y\right),0.4e^{\left(0.3\right)2\pi i}0.2e^{\left(0.6\right)2\pi i}\right) \end{array}\right\}$$

*Then, the complex intuitionistic composite fuzzy relation* $R_{1}\circ R_{2}$ *is given by*

$$R_{1}\circ R_{2}=\left\{\begin{array}{c} \left(\left(x,x\right),0.3e^{\left(0.4\right)2\pi i}0.5e^{\left(0.5\right)2\pi i}\right),\left(\left(y,x\right),0.3e^{\left(0.4\right)2\pi i}0.5e^{\left(0.5\right)2\pi i}\right),\\ \left(\left(z,y\right),0.4e^{\left(0.3\right)2\pi i}0.2e^{\left(0.6\right)2\pi i}\right) \end{array}\right\}$$

**Theorem** **1.** *The CIFR*R*is a complex intuitionistic symmetric FR on a CIFS*Ḯ $iff\;R=R^{c}$.

**Proof.**  Let $R=R^{c}$, then

$$\left(u,v\right)\in R\Leftrightarrow \left(v,u\right)\in R^{c}\Leftrightarrow \left(v,u\right)\in R$$

Hence, R is a complex intuitionistic symmetric FR on a CIFS Ḯ.

Conversely, suppose that R is a complex intuitionistic symmetric FR on a CIFS Ḯ, then

(u,v)∈R⇔(v,u)∈R

However, $\left(v,u\right)\in R^{c}\Rightarrow R=R^{c}$.  □

**Theorem** **2.** *The CIFR*ℛ*is a complex intuitionistic transitive FR on a CIFS*Ḯ iff R∘R⊆R.

**Proof.**  Let R is a complex intuitionistic transitive FR on a CIFS Ḯ. Assume that

(u,w)∈R∘R

then, by the transitivity of R,

(u,v)∈R and (v,w)∈R⇒(u,w)∈R⇒R∘R⊆R.

Conversely, suppose that R∘R⊆R; then, for

(u,v)∈R and (v,w)∈R⇒(u,w)∈R∘R⊆R⇒(u,w)∈R.

Hence, R is complex intuitionistic transitive FR on Ḯ.  □

**Theorem** **3.** *If*R*is a complex intuitionistic equivalence FR on a CIFS*Ḯ, *then*R∘R=R.

**Proof.**  Let (u,v)∈R

Then, by the symmetry of a complex intuitionistic equivalence FR R,

(v,u)∈R

Now, by using the transitive property of a complex intuitionistic equivalence FR R,

(u,u)∈R

However, by the definition of complex intuitionistic composite FR,

(u,u)∈R∘R

(1) Thus R⊆R∘R.

Conversely, suppose that (u,v)∈R∘R, then ∃ w∈U∋(u,w)∈R and (w,v)∈R However, since R is a complex intuitionistic equivalence FR on Ḯ, R is also a complex intuitionistic transitive FR. Thus,

(2) (u,v)∈R⇒R∘R⊆R

Hence, by (1) and (2),

R∘R=R.

 □

**Theorem** **4.** *The converse of a complex intuitionistic partial order FR*R*on a CIFS*Ḯ*is also a complex intuitionistic partial order FR on*Ḯ.

**Proof.**  In order to prove the assertion, it is sufficient to show that the converse of a complex intuitionistic partial order FR $R^{c}$ satisfies the three properties of a complex intuitionistic partial order FR.

i Since R is a complex intuitionistic reflexive FR. Thus, for some u∈U,
  $$\left(u,u\right)\in R\Rightarrow \left(u,u\right)\in R^{c}$$

Hence, ${ℛ}^{c}$ is a complex intuitionistic reflexive FR.

ii Let $\left(u,u\right)\in R^{c}$ and $\left(v,u\right)\in R^{c}$
  Then (u,v)∈R and (v,u)∈R

However, R is a complex intuitionistic anti-symmetric FR. Thus,

(u,v)=(v,u)

Therefore, $R^{c}$ is also a complex intuitionistic anti-symmetric FR.

iii Let $\left(u,v\right)\in R^{c}$ and $\left(v,w\right)\in R^{c}$
  Then (w,v)∈R and (v,u)∈R

However, since R is a complex intuitionistic transitive FR. Thus,

$$\left(w,u\right)\in R\Rightarrow \left(u,w\right)\in R^{c}.$$

Thus, $R^{c}$ is also a complex intuitionistic transitive FR.

From i, ii and iii, it is proven that $R^{c}$ is also a complex intuitionistic partial order FR.  □

**Theorem** **5.** *If*R*is a complex intuitionistic equivalence FR on a CIFS*Ḯ, *then*(u,v)∈R,  iff R[u]=R[v].

**Proof.**  Let (u,v)∈R and w∈R[u]⇒(w,u)∈R.

Now, by the transitive property of R,

(3) (w,v)∈R⇒w∈R[v].

Therefore, R[u]⊆R[v].

Since (u,v)∈R, by using the symmetric property of R

(v,u)∈R.

Moreover, suppose that w∈R[v]⇒(w,v)∈R.

Now, by the transitive property of R,

(4) (w,u)∈R⇒w∈R[u].

Therefore, R[v]⊆R[u].

Hence, (3) and (4) imply that R[v]=R[u].

Conversely, let R[v]=R[u], w∈R[u] and w∈R[v]⇒(w,v)∈R and (w,u)∈R Using the symmetric property of R

(w,u)∈R⇒(u,w)∈R

Now, by the transitive property of R, (u,w)∈R and (w,v)∈R⇒(u,v)∈R, which completes the proof. □

**Definition** **13.** *A pictorial delineation of the partial order relation is called a Hasse diagram. It consists of dots and line segments known as vertices and edges, respectively. A Hasse diagram does not have self-loops but directional and redundant edges. Each element of the set is represented by a vertex and the relationship among them is represented through edges that join these vertices according to the following rules*:

- *The elements are arranged in an order or ranked up and down based on their relationships. The elements that are related to all other elements are kept at the bottom and the elements to whom every element of the set is related are kept at the top*.
- *Two vertices are joined by an edge if and only if they are related to each other*.

**Example** **11.** *Let us draw a Hasse diagram of the complex intuitionistic partial order FR*R*on a CIFS*$Ḯ=\left\{\begin{array}{c} \begin{array}{c} \left(p,0.31e^{\left(0.52\right)2\pi i},0.53e^{\left(0.44\right)2\pi i}\right),\left(q,0.65e^{\left(0.76\right)2\pi i},0.27e^{\left(0.18\right)2\pi i}\right) \end{array},\\ \left(r,0.29e^{\left(0.40\right)2\pi i},0.61e^{\left(0.52\right)2\pi i}\right),\left(s,0.13e^{\left(0.44\right)2\pi i},0.15e^{\left(0.36\right)2\pi i}\right),\\ \left(t,0.67e^{\left(0.18\right)2\pi i},0.12e^{\left(0.80\right)2\pi i}\right) \end{array}\right\}$,

*The CP is found to be*

$$Ḯ\times Ḯ=\{\begin{array}{c} \left(\left(p,p\right),0.31e^{\left(0.52\right)2\pi i},0.53e^{\left(0.44\right)2\pi i}\right),\left(\left(p,q\right),0.31e^{\left(0.52\right)2\pi i},0.53e^{\left(0.44\right)2\pi i}\right),\\ \left(\left(p,r\right),0.29e^{\left(0.40\right)2\pi i},0.61e^{\left(0.52\right)2\pi i}\right),\left(\left(p,s\right),0.13e^{\left(0.44\right)2\pi i},0.53e^{\left(0.44\right)2\pi i}\right),\\ \left(\left(p,t\right),0.31e^{\left(0.18\right)2\pi i},0.53e^{\left(0.80\right)2\pi i}\right),\left(\left(q,p\right),0.31e^{\left(0.52\right)2\pi i},0.53e^{\left(0.44\right)2\pi i}\right),\\ \left(\left(q,q\right),0.65e^{\left(0.76\right)2\pi i},0.27e^{\left(0.18\right)2\pi i}\right),\left(\left(q,r\right),0.29e^{\left(0.40\right)2\pi i},0.61e^{\left(0.52\right)2\pi i}\right),\\ \left(\left(q,s\right),0.13e^{\left(0.44\right)2\pi i},0.27e^{\left(0.18\right)2\pi i}\right),\left(\left(q,t\right),0.67e^{\left(0.18\right)2\pi i},0.27e^{\left(0.80\right)2\pi i}\right),\\ \left(\left(r,p\right),0.29e^{\left(0.40\right)2\pi i},0.61e^{\left(0.52\right)2\pi i}\right),\left(\left(r,q\right),0.29e^{\left(0.40\right)2\pi i},0.61e^{\left(0.52\right)2\pi i}\right),\\ \left(\left(r,r\right),0.29e^{\left(0.40\right)2\pi i},0.61e^{\left(0.52\right)2\pi i}\right),\left(\left(r,s\right),0.13e^{\left(0.40\right)2\pi i},0.61e^{\left(0.52\right)2\pi i}\right),\\ \left(\left(r,t\right),0.29e^{\left(0.18\right)2\pi i},0.61e^{\left(0.80\right)2\pi i}\right),\left(\left(s,p\right),0.13e^{\left(0.44\right)2\pi i},0.53e^{\left(0.44\right)2\pi i}\right),\\ \left(\left(s,q\right),0.13e^{\left(0.44\right)2\pi i},0.27e^{\left(0.18\right)2\pi i}\right),\left(\left(s,r\right),0.13e^{\left(0.40\right)2\pi i},0.61e^{\left(0.52\right)2\pi i}\right),\\ \left(\left(s,s\right),0.13e^{\left(0.44\right)2\pi i},0.15e^{\left(0.36\right)2\pi i}\right),\left(\left(s,t\right),0.13e^{\left(0.18\right)2\pi i},0.15e^{\left(0.80\right)2\pi i}\right),\\ \left(\left(t,p\right),0.31e^{\left(0.18\right)2\pi i},0.53e^{\left(0.80\right)2\pi i}\right),\left(\left(t,q\right),0.67e^{\left(0.18\right)2\pi i},0.27e^{\left(0.80\right)2\pi i}\right),\\ \left(\left(t,r\right),0.29e^{\left(0.18\right)2\pi i},0.61e^{\left(0.80\right)2\pi i}\right),\left(\left(t,s\right),0.13e^{\left(0.18\right)2\pi i},0.15e^{\left(0.80\right)2\pi i}\right),\\ \left(\left(t,t\right),0.67e^{\left(0.18\right)2\pi i},0.12e^{\left(0.80\right)2\pi i}\right) \end{array}\}$$

*A complex intuitionistic partial order FR* R *is*

$$R=\left\{\begin{array}{c} \left(\left(p,p\right),0.31e^{\left(0.52\right)2\pi i},0.53e^{\left(0.44\right)2\pi i}\right),\left(\left(p,q\right),0.31e^{\left(0.52\right)2\pi i},0.53e^{\left(0.44\right)2\pi i}\right),\\ \left(\left(p,r\right),0.29e^{\left(0.40\right)2\pi i},0.61e^{\left(0.52\right)2\pi i}\right),\left(\left(p,s\right),0.13e^{\left(0.44\right)2\pi i},0.53e^{\left(0.44\right)2\pi i}\right),\\ \left(\left(p,t\right),0.31e^{\left(0.18\right)2\pi i},0.53e^{\left(0.80\right)2\pi i}\right),\left(\left(q,q\right),0.65e^{\left(0.76\right)2\pi i},0.27e^{\left(0.18\right)2\pi i}\right),\\ \left(\left(q,s\right),0.13e^{\left(0.44\right)2\pi i},0.27e^{\left(0.18\right)2\pi i}\right),\left(\left(q,t\right),0.67e^{\left(0.18\right)2\pi i},0.27e^{\left(0.80\right)2\pi i}\right),\\ \left(\left(r,q\right),0.29e^{\left(0.40\right)2\pi i},0.61e^{\left(0.52\right)2\pi i}\right),\left(\left(r,r\right),0.29e^{\left(0.40\right)2\pi i},0.61e^{\left(0.52\right)2\pi i}\right),\\ \left(\left(r,s\right),0.13e^{\left(0.40\right)2\pi i},0.61e^{\left(0.52\right)2\pi i}\right),\left(\left(r,t\right),0.29e^{\left(0.18\right)2\pi i},0.61e^{\left(0.80\right)2\pi i}\right),\\ \left(\left(s,s\right),0.13e^{\left(0.44\right)2\pi i},0.15e^{\left(0.36\right)2\pi i}\right),\left(\left(s,t\right),0.13e^{\left(0.18\right)2\pi i},0.15e^{\left(0.80\right)2\pi i}\right),\\ \left(\left(t,t\right),0.67e^{\left(0.18\right)2\pi i},0.12e^{\left(0.80\right)2\pi i}\right) \end{array}\right\}$$

**Figure 3:**
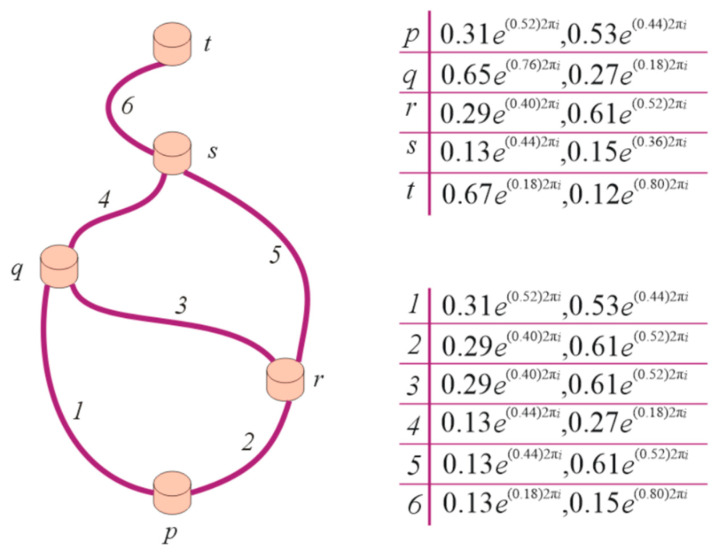
Hasse diagram for R.

*Figure 3 displays the Hasse diagram of*R.

**Definition** **14.** *An element*

1. *That succeeds all the other elements is known as the maximum or greatest element*.
2. *That precedes all the other elements is known as the minimum or least element*.
3. *That is not related to any other element is known as the maximal element. The topmost elements of the Hasse diagram are the maximal elements*.
4. *To whom any other element(s) is(are) not related is(are) known as the minimal element(s). In other words, the element(s) that is(are) related to every other element is(are) the minimal element(s). The bottommost elements of the Hasse diagram are the minimal elements*.

**Figure 4:**
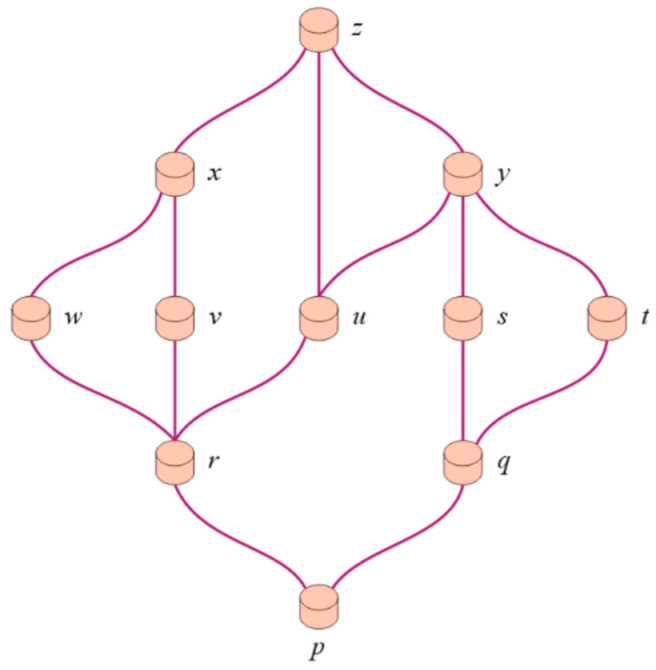
Hasse diagram for maximum, maximal, minimum and minimal elements.

**Example** **12.** *Let*{p,q,r,s,t,u,v,w,x,y,z}*be the elements of a complex intuitionistic partial order fuzzy set*Ḯ. *For convenience, we ignore the membership and non-membership grades. Figure 4 shows the Hasse diagram of set*Ḯ.

*In the above diagram*, z*is the maximum and maximal element, while*p*is the minimum and minimal element*.

**Definition** **15.** *For a subset*ʝ*of*Ḯ, *an element*u∈R⊆Ḯ×Ḯ*is known as the*

1. *upper bound of*ʝ*if*(v,u)∈R,  ∀v∈ʝ.
2. *lower bound of*ʝ*if*(u,v)∈R,  ∀v∈ʝ.

**Definition** **16.** *Let*ʝ*be a subset of a CIFS*Ḯ, *then the least upper bound and the greatest lower bound of*ʝ*are called the supremum and infimum of*ʝ, *respectively*.

**Figure 5:**
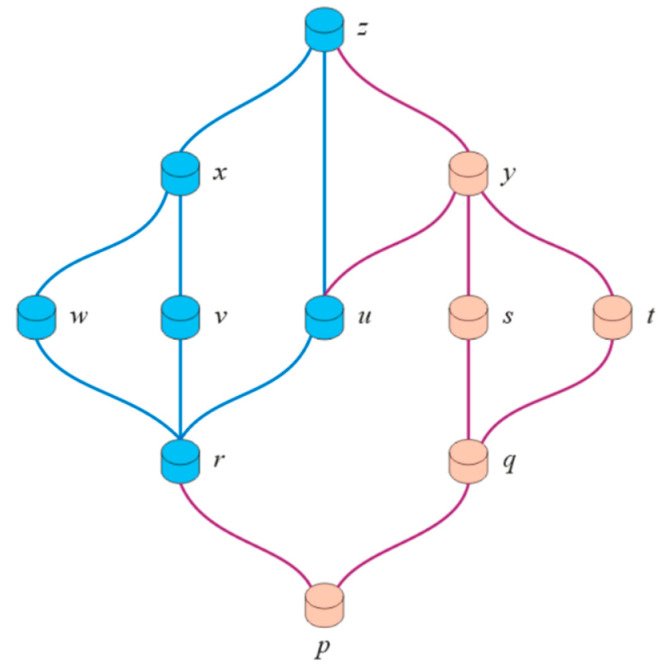
Hasse diagram for upper and lower bounds, supremum and infimum.

**Example** **13.** *Let*{p,q,r,s,t,u,v,w,x,y,z}*be the elements of a complex intuitionistic partial order fuzzy set*Ḯ*and*ʝ={q,r,s,t,u,y}*be a subset of*Ḯ. *For convenience, we ignore the membership and non-membership grades. Figure 5 shows the Hasse diagram of set*ʝ. *The elements of set*Ḯ*are colored blue*.

*In the above diagram*, y*and*z*are the upper bounds of*ʝ, *whereas*, y*is the supremum of*ʝ.

*On the other hand*, p*and*r*are the lower bound of*ʝ, *while*r*is the infimum of*ʝ.

## 5. Application

This section presents a couple of applications of the proposed concepts in the fields of information technology; more specifically, we consider cyber-security and cyber-crimes in the oil and gas industries.

### 5.1. Cyber-Security in Oil and Gas Industries

**Figure 6:**
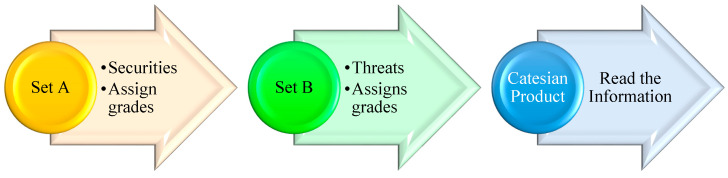
Flowchart for the process being followed.

Huge development and modernization has taken place as a result of digitalization among various industries, and the oil and gas sector is no exception. Although advanced technological solutions such as IIOT (Industrial Internet of Things) have improved efficiency and reduced industrial expenditures, they have also exposed the oil and gas industries to the risks of cyber-crimes. These threats can have severely negative impacts on the company, resulting in massive losses of money and reputation and leading to environmental disasters. Below are some threats and the methods used for security purposes by an oil and gas company. Figure 6 presents the flowchart for the process followed in the application.

#### 5.1.1. Threats

Some of the threats that an oil and gas company are vulnerable to are explained below. Moreover, each threat and malware has been assigned the membership and non-membership grades. These membership grades are set by professionals according to their performance and operation. The membership grade for a threat indicates its weakness, while the non-membership grade shows the strength or the severity of the threat. Since the grades range between 0 and 1, in the case of the membership grade, values closer to 1 represent greater effectiveness or success in accomplishing the target, while lower values i.e., close to zero, indicate less effectiveness and success in accomplishing the target. On the other hand, higher values of the non-membership grade indicate a higher likelihood of failure in achieving the goals and vice versa. Table 1 summarizes this section. The amplitude term represents the level of strength or weakness, while the phase term refers to the timeframe. In addition, higher values of the phase terms reflect a greater time period and the lower values refer to a shorter timespan.

- Infrastructure sabotage (IS): Cyber-criminals deploy malware and viruses that are specifically used to sabotage the networks, control systems or servers. The oil and gas industries have been attacked by different types of wiper malwares, such as Stuxnet malware and Industroyer.
  $$\left(IS,\;0.4e^{\left(0.5\right)\pi i},0.5e^{\left(0.5\right)\pi i}\right)$$
- Espionage and data theft (E&DT) are very serious concerns. Industries and companies are highly dependent on unique information that keeps them ahead of their competitors. In the oil and gas sector, data such as experimental results, boring procedures, new oil reserves and the chemistry of top products are extremely valuable. Thus, such data carry the greatest risk. Some tactics used for such attacks include DNS hijacking, phishing emails and corporate VPN servers or even scraping information that is openly available to obtain data.
  $$\left(E\&DT,\;0.5e^{\left(0.6\right)\pi i},0.5e^{\left(0.2\right)\pi i}\right)$$
- Ever-changing malware (ECM): Usually, there are different malwares that are executed to fulfil different purposes, such as intrusion, data theft, propagation, etc. A cyber-criminal wishes to maintain their access to the targeted system in order to steal critical information by continuously and constantly updating the malware codes. Some malwares used by cyber-criminals to infect targets, maintain persistence and communicate are web-shells, DNS tunneling, email and cloud services.
  $$\left(ECM,\;0.4e^{\left(0.4\right)\pi i},0.6e^{\left(0.5\right)\pi i}\right)$$
- Ransomware (RW) is a malware that is used to steal or encode the valuable information of a company. These malwares hugely impact the regular operations of an industry. In order to recover lost data, the industries are more likely to pay the ransom.
  $$\left(RW,\;0.6e^{\left(0.7\right)\pi i},0.4e^{\left(0.2\right)\pi i}\right)$$
- Insider threat (InT) is a serious threat to an industry that comes from the employees, former employees, contractors or business associates of the industry, i.e., people with confidential information about security practices, data and the digital system.
  $$\left(InT,0.4e^{\left(0.3\right)\pi i},0.6e^{\left(0.6\right)\pi i}\right)$$

Henceforth, the CIFS ʝ summarizing the security threats is given below:

$$ʝ=\left\{\begin{array}{c} \left(IS,\;0.4e^{\left(0.5\right)\pi i},0.5e^{\left(0.5\right)\pi i}\right),\left(E\&DT,\;0.5e^{\left(0.6\right)\pi i},0.5e^{\left(0.2\right)\pi i}\right),\\ \left(ECM,\;0.4e^{\left(0.4\right)\pi i},0.6e^{\left(0.5\right)\pi i}\right),\left(RW,\;0.6e^{\left(0.7\right)\pi i},0.4e^{\left(0.2\right)\pi i}\right),\\ \left(InT,0.4e^{\left(0.3\right)\pi i},0.6e^{\left(0.6\right)\pi i}\right) \end{array}\right\}$$

#### 5.1.2. Security Methods

The methods, techniques and practices adopted by the oil and gas industries to protect against cyber-crimes are discussed below. Each security method is assigned a pair of functions in the form of membership and non-membership grades. The membership grade represents the security level of a method, while the non-membership grade represents the risk levels that can result from implementing these techniques. Table 2 recaps this section. The amplitude term refers to the level or grade of security or risk, while the phase term refers to time. The assignation of the values the phase terms and amplitude term of membership and non-membership grades is similar to the assignment of values to the threats. Obviously, greater values of amplitude terms of membership grades are preferable with regard to security because they indicate greater security. However, higher values of phase terms of membership grades are also important, as they indicate long-term security. On the contrary, smaller values of amplitude terms of non-membership grades refer to a lower risk or insecurity. Thus, better security is indicated by a greater amplitude term value as well as a greater phase term value of its membership grade and lower values of the amplitude term and phase term of non-membership grades. Some of the most important security methods for the oil and gas industry are defined below with their fuzzy grades.

- Deep Armor Industrial (DMI) is an AI-based technology that identifies and reports new devices or irregular activities such as insider threats and digital–physical attacks. Due to its predictive analysis, the execution of malicious codes is prevented. It is highly effective and can stop codes that are not yet present in threat intelligence packages. DMI provides unique, innovative and exceptional security to the oil and gas industries, even against new threats that emerge between updates, or cyber-attacks that arrive at isolated sites before patches can be deployed. It intends to provide the latest antivirus software, detection of threats, control of application and zero-day attack prevention to the industry.
  $$\left(DMI,\;0.7e^{\left(0.8\right)\pi i},0.1e^{\left(0.2\right)\pi i}\right)$$
- ABB’s Event Monitoring (EM) is a cyber-security solution that enables the security teams to more effectively identify, rank and react to threats across an OT network. Consecutively, it aids in the mitigation of risks significantly. Moreover, it eliminates the manual monitoring tasks, which saves the time in the process of threat detection. Thus, the customers can focus on value-added tasks.
  $$\left(EM,\;0.6e^{\left(0.6\right)\pi i},0.3e^{\left(0.3\right)\pi i}\right)$$
- ABB’s cyber-security workplace (CSW) is another innovative solution that enables companies to automatically and securely perform maintenance and routine tasks for their plant, without affecting the safety. It also offers the control and tracking of security patches, backup frequency and critical hardening measures.
  $$\left(CSW,\;0.6e^{\left(0.4\right)\pi i},0.2e^{\left(0.4\right)\pi i}\right)$$
- Nozomi network solution (NNS) is an innovative cyber-security solution that provides greater threat detection, accurate asset discovery and flexible and scalable deployment. It also provides superior operational visibility and OT cyber-security.
  $$\left(NNS,\;0.5e^{\left(0.5\right)\pi i},0.3e^{\left(0.4\right)\pi i}\right)$$
- Forge Cyber-Security Suite (FCS) is a strong software solution that is used by the oil and gas industries to simplify, strengthen and scale the industrial cyber-security operations when facing evolving threats.
  $$\left(FCS,\;0.5e^{\left(0.4\right)\pi i},0.2e^{\left(0.4\right)\pi i}\right)$$
- Managed Security Services (MSS) proactively monitors, measures and manages industrial cyber-security risk.
  $$\left(MSS,\;0.4e^{\left(0.5\right)\pi i},0.4e^{\left(0.2\right)\pi i}\right)$$

Henceforth, the following CIFS Ḯ summarizing the security methods is constructed:

$$Ḯ=\left\{\begin{array}{c} \left(DMI,\;0.7e^{\left(0.8\right)\pi i},0.1e^{\left(0.2\right)\pi i}\right),\left(EM,\;0.6e^{\left(0.6\right)\pi i},0.3e^{\left(0.3\right)\pi i}\right),\\ \left(CSW,\;0.6e^{\left(0.4\right)\pi i},0.2e^{\left(0.4\right)\pi i}\right),\left(NNS,\;0.5e^{\left(0.5\right)\pi i},0.3e^{\left(0.4\right)\pi i}\right),\\ \left(FCS,\;0.5e^{\left(0.4\right)\pi i},0.2e^{\left(0.4\right)\pi i}\right),\left(MSS,\;0.4e^{\left(0.5\right)\pi i},0.4e^{\left(0.2\right)\pi i}\right) \end{array}\right\}$$

#### 5.1.3. Calculations

Here, in order to study the relationships among the effectiveness and incompetency of each cyber-security technique against every cyber-crime, we carry out the following mathematics.

We have the following two CIFSs Ḯ and ʝ representing the set of securities and the set of threats, respectively.

$$Ḯ=\left\{\begin{array}{c} \left(DMI,\;0.7e^{\left(0.8\right)\pi i},0.1e^{\left(0.2\right)\pi i}\right),\left(EM,\;0.6e^{\left(0.6\right)\pi i},0.3e^{\left(0.3\right)\pi i}\right),\\ \left(CSW,\;0.6e^{\left(0.4\right)\pi i},0.2e^{\left(0.4\right)\pi i}\right),\left(NNS,\;0.5e^{\left(0.5\right)\pi i},0.3e^{\left(0.4\right)\pi i}\right),\\ \left(FCS,\;0.5e^{\left(0.4\right)\pi i},0.2e^{\left(0.4\right)\pi i}\right),\left(MSS,\;0.4e^{\left(0.5\right)\pi i},0.4e^{\left(0.2\right)\pi i}\right) \end{array}\right\}$$

$$ʝ=\left\{\begin{array}{c} \left(IS,\;0.4e^{\left(0.5\right)\pi i},0.5e^{\left(0.5\right)\pi i}\right),\left(E\&DT,\;0.5e^{\left(0.6\right)\pi i},0.5e^{\left(0.2\right)\pi i}\right),\\ \left(ECM,\;0.4e^{\left(0.4\right)\pi i},0.6e^{\left(0.5\right)\pi i}\right),\left(RW,\;0.6e^{\left(0.7\right)\pi i},0.4e^{\left(0.2\right)\pi i}\right),\\ \left(InT,0.4e^{\left(0.3\right)\pi i},0.6e^{\left(0.6\right)\pi i}\right) \end{array}\right\}$$

With the intention of determining the effectiveness of the security methods against each threat, we calculate the CP Ḯ×ʝ. By using Definition 8, we have


$$Ḯ\times ʝ=\left\{\begin{array}{c} \left(\left(DMI,IS\right),0.4e^{\left(0.5\right)\pi i},0.5e^{\left(0.5\right)\pi i}\right),\left(\left(DMI,E\&DT\right),0.5e^{\left(0.6\right)\pi i},0.5e^{\left(0.2\right)\pi i}\right),\\ \left(\left(DMI,ECM\right),0.4e^{\left(0.4\right)\pi i},0.2e^{\left(0.4\right)\pi i}\right),\left(\left(DMI,RW\right),0.6e^{\left(0.7\right)\pi i},0.4e^{\left(0.2\right)\pi i}\right),\\ \left(\left(DMI,InT\right),0.4e^{\left(0.3\right)\pi i},0.6e^{\left(0.6\right)\pi i}\right),\left(\left(EM,IS\right),0.4e^{\left(0.5\right)\pi i},0.5e^{\left(0.5\right)\pi i}\right),\\ \left(\left(EM,E\&DT\right),0.5e^{\left(0.6\right)\pi i},0.5e^{\left(0.3\right)\pi i}\right),\left(\left(EM,ECM\right),0.4e^{\left(0.4\right)\pi i},0.6e^{\left(0.5\right)\pi i}\right),\\ \left(\left(EM,RW\right),0.6e^{\left(0.6\right)\pi i},0.4e^{\left(0.3\right)\pi i}\right),\left(\left(EM,InT\right),0.4e^{\left(0.3\right)\pi i},0.6e^{\left(0.6\right)\pi i}\right),\\ \left(\left(CSW,IS\right),0.4e^{\left(0.4\right)\pi i},0.5e^{\left(0.5\right)\pi i}\right),\left(\left(CSW,E\&DT\right),0.5e^{\left(0.4\right)\pi i},0.5e^{\left(0.4\right)\pi i}\right),\\ \left(\left(CSW,ECM\right),\;0.4e^{\left(0.4\right)\pi i},0.6e^{\left(0.5\right)\pi i}\right),\left(\left(CSW,RW\right),\;0.6e^{\left(0.4\right)\pi i},0.4e^{\left(0.4\right)\pi i}\right),\\ \left(\left(CSW,InT\right),0.4e^{\left(0.3\right)\pi i},0.6e^{\left(0.6\right)\pi i}\right),\left(\left(NNS,IS\right),0.4e^{\left(0.5\right)\pi i},0.5e^{\left(0.5\right)\pi i}\right),\\ \left(\left(NNS,E\&DT\right),\;0.5e^{\left(0.5\right)\pi i},0.5e^{\left(0.4\right)\pi i}\right),\left(\left(NNS,ECM\right),0.4e^{\left(0.4\right)\pi i},0.6e^{\left(0.5\right)\pi i}\right),\\ \left(\left(NNS,RW\right),\;0.5e^{\left(0.5\right)\pi i},0.4e^{\left(0.4\right)\pi i}\right),\left(\left(NNS,InT\right),0.4e^{\left(0.3\right)\pi i},0.6e^{\left(0.6\right)\pi i}\right),\\ \left(\left(FCS,IS\right),\;0.4e^{\left(0.4\right)\pi i},0.5e^{\left(0.5\right)\pi i}\right),\left(\left(FCS,E\&DT\right),0.5e^{\left(0.4\right)\pi i},0.5e^{\left(0.4\right)\pi i}\right),\\ \left(\left(FCS,ECM\right),0.4e^{\left(0.4\right)\pi i},0.6e^{\left(0.5\right)\pi i}\right),\left(\left(FCS,RW\right),\;0.5e^{\left(0.4\right)\pi i},\;0.4e^{\left(0.4\right)\pi i}\right),\\ \left(\left(FCS,InT\right),0.4e^{\left(0.3\right)\pi i},0.6e^{\left(0.6\right)\pi i}\right),\left(\left(MSS,IS\right),0.4e^{\left(0.5\right)\pi i},0.5e^{\left(0.5\right)\pi i}\right),\\ \left(\left(MSS,E\&DT\right),\;0.4e^{\left(0.5\right)\pi i},0.5e^{\left(0.2\right)\pi i}\right),\left(\left(MSS,ECM\right),0.4e^{\left(0.4\right)\pi i},0.6e^{\left(0.5\right)\pi i}\right),\\ \left(\left(MSS,RW\right),\;0.4e^{\left(0.5\right)\pi i},0.4e^{\left(0.2\right)\pi i}\right),\left(\left(MSS,InT\right),0.4e^{\left(0.3\right)\pi i},0.6e^{\left(0.6\right)\pi i}\right) \end{array}\right\}$$

**Figure 7:**
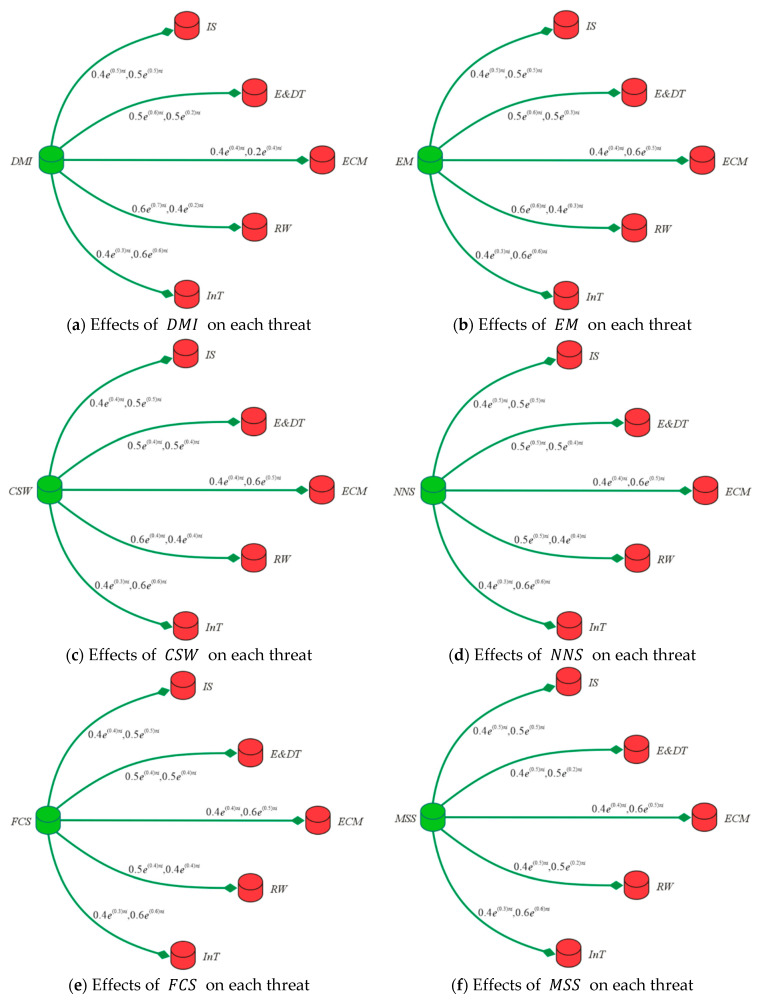
Effects of different cyber-security measures on each threat.

Each element of  Ḯ×ʝ is in the form of an order pair, which represents the relationship among the pair, i.e., the effects and impacts of the first term on the second term in the pair. The membership grades indicate the effectiveness of a security technique to eliminate a particular threat with respect to some time unit. On the other hand, the non-membership grades reflect the uselessness or ineffectiveness of a certain security method against a specific threat. For example, the element $\left(\left(EM,RW\right),0.6e^{\left(0.6\right)\pi i},0.4e^{\left(0.3\right)\pi i}\right)$ conveys that the event monitoring solution can effectively resolve the ransomware and the level of ineffectiveness is low. The numbers translate as follows: the level of security of event monitoring solution against the ransomware is 0.6 with respect to 0.6 time units and the level of risk due to ransomware after applying the event monitoring solution is 0.4 with respect to 0.3 time units. Regarding the security, a greater timeframe in the membership grade is better, while a shorter time in the non-membership grade is better. The diagrams in Figure 7 demonstrate the above relationships.

### 5.2. Selection of the Cyber-Security Techniques

**Figure 8:**
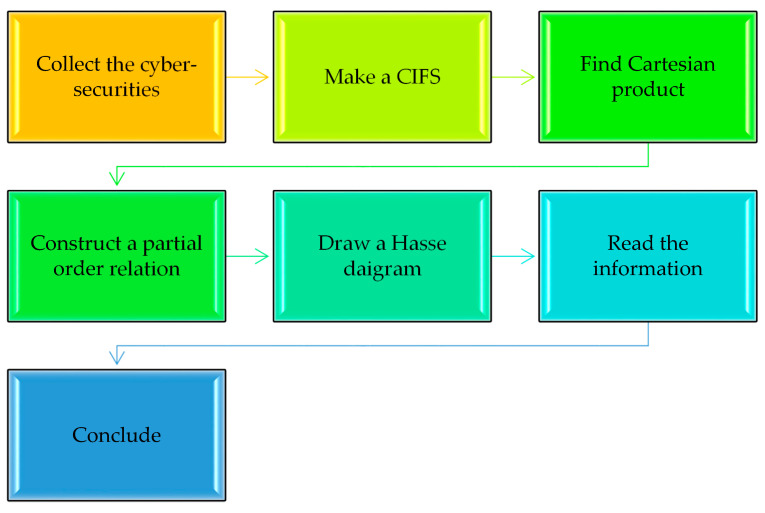
Flowchart of the process for selecting the best cyber-security measure.

Suppose that an enterprise or company wishes to implement some cyber-security techniques in order to control and counter the potential threats and reduce the risks of cyber-attacks. There are certain possible techniques, which are given in the following Table 3. However, the company needs to select the best possible techniques that would resolve the issues that are being faced. The complete process of this application is shown in Figure 8.

Let us assign the supposed membership and non-membership grades to each of the security measures and construct a CIFS Ḯ:

$$Ḯ=\left\{\begin{array}{c} \left(DMI,\;0.7e^{\left(0.8\right)\pi i},0.1e^{\left(0.2\right)\pi i}\right),\left(EM,\;0.6e^{\left(0.6\right)\pi i},0.3e^{\left(0.3\right)\pi i}\right),\\ \left(CSW,\;0.6e^{\left(0.4\right)\pi i},0.2e^{\left(0.4\right)\pi i}\right),\left(NNS,\;0.5e^{\left(0.5\right)\pi i},0.3e^{\left(0.4\right)\pi i}\right),\\ \left(FCS,\;0.5e^{\left(0.4\right)\pi i},0.2e^{\left(0.4\right)\pi i}\right),\left(MSS,\;0.4e^{\left(0.5\right)\pi i},0.4e^{\left(0.2\right)\pi i}\right),\\ \left(BC,\;0.8e^{\left(0.8\right)\pi i},0.2e^{\left(0.1\right)\pi i}\right),\left(FW,\;0.5e^{\left(0.6\right)\pi i},0.1e^{\left(0.2\right)\pi i}\right),\\ \left(SSW,\;0.3e^{\left(0.4\right)\pi i},0.5e^{\left(0.6\right)\pi i}\right) \end{array}\right\}$$

Based on the CP Ḯ×Ḯ (using Definition 8), a complex intuitionistic partial order FR R (Definition 9) is obtained:

$$R=\left\{\begin{array}{c} \left(\left(DMI,DMI\right),0.7e^{\left(0.8\right)\pi i},0.1e^{\left(0.2\right)\pi i}\right),\left(\left(DMI,BC\right),0.7e^{\left(0.8\right)\pi i},0.2e^{\left(0.2\right)\pi i}\right),\\ \left(\left(EM,EM\right),0.6e^{\left(0.6\right)\pi i},0.3e^{\left(0.3\right)\pi i}\right),\left(\left(EM,BC\right),0.6e^{\left(0.6\right)\pi i},0.3e^{\left(0.3\right)\pi i}\right),\\ \left(\left(CSW,EM\right),0.6e^{\left(0.4\right)\pi i},0.3e^{\left(0.4\right)\pi i}\right),\left(\left(CSW,CSW\right),0.6e^{\left(0.4\right)\pi i},0.2e^{\left(0.4\right)\pi i}\right),\\ \left(\left(CSW,BC\right),0.6e^{\left(0.4\right)\pi i},0.2e^{\left(0.4\right)\pi i}\right),\left(\left(NNS,DMI\right),\;0.5e^{\left(0.5\right)\pi i},0.3e^{\left(0.4\right)\pi i}\right),\\ \left(\left(NNS,EM\right),\;0.5e^{\left(0.5\right)\pi i},0.3e^{\left(0.4\right)\pi i}\right),\left(\left(NNS,NNS\right),\;0.5e^{\left(0.5\right)\pi i},0.3e^{\left(0.4\right)\pi i}\right),\\ \left(\left(NNS,BC\right),\;0.5e^{\left(0.5\right)\pi i},0.3e^{\left(0.4\right)\pi i}\right),\left(\left(FCS,DMI\right),\;0.5e^{\left(0.4\right)\pi i},0.2e^{\left(0.4\right)\pi i}\right),\\ \left(\left(FCS,EM\right),\;0.5e^{\left(0.4\right)\pi i},0.3e^{\left(0.4\right)\pi i}\right),\left(\left(FCS,NNS\right),\;0.5e^{\left(0.4\right)\pi i},0.3e^{\left(0.4\right)\pi i}\right),\\ \left(\left(FCS,FCS\right),\;0.5e^{\left(0.4\right)\pi i},0.2e^{\left(0.4\right)\pi i}\right),\left(\left(FCS,FW\right),\;0.5e^{\left(0.4\right)\pi i},0.2e^{\left(0.4\right)\pi i}\right),\\ \left(\left(FCS,BC\right),\;0.5e^{\left(0.4\right)\pi i},0.2e^{\left(0.4\right)\pi i}\right),\left(\left(MSS,EM\right),0.4e^{\left(0.5\right)\pi i},0.4e^{\left(0.3\right)\pi i}\right),\\ \left(\left(MSS,CSW\right),0.4e^{\left(0.5\right)\pi i},0.4e^{\left(0.4\right)\pi i}\right),\left(\left(MSS,MSS\right),\;0.4e^{\left(0.5\right)\pi i},0.4e^{\left(0.2\right)\pi i}\right),\\ \left(\left(MSS,BC\right),\;0.4e^{\left(0.5\right)\pi i},0.4e^{\left(0.2\right)\pi i}\right),\left(\left(BC,BC\right),0.8e^{\left(0.8\right)\pi i},0.2e^{\left(0.1\right)\pi i}\right),\\ \left(\left(FW,DMI\right),0.5e^{\left(0.6\right)\pi i},0.1e^{\left(0.2\right)\pi i}\right),\left(\left(FW,EM\right),0.5e^{\left(0.6\right)\pi i},0.3e^{\left(0.3\right)\pi i}\right),\\ \left(\left(FW,NNS\right),0.5e^{\left(0.5\right)\pi i},0.3e^{\left(0.4\right)\pi i}\right),\left(\left(FW,FW\right),0.5e^{\left(0.6\right)\pi i},0.1e^{\left(0.2\right)\pi i}\right),\\ \left(\left(FW,BC\right),0.5e^{\left(0.6\right)\pi i},0.2e^{\left(0.2\right)\pi i}\right),\left(\left(SSW,DMI\right),0.3e^{\left(0.4\right)\pi i},0.5e^{\left(0.6\right)\pi i}\right),\\ \left(\left(SSW,EM\right),0.3e^{\left(0.4\right)\pi i},0.5e^{\left(0.6\right)\pi i}\right),\left(\left(SSW,CSW\right),0.3e^{\left(0.4\right)\pi i},0.5e^{\left(0.6\right)\pi i}\right),\\ \left(\left(SSW,NNS\right)0.3e^{\left(0.4\right)\pi i},0.5e^{\left(0.6\right)\pi i}\right),\left(\left(SSW,FCS\right),0.3e^{\left(0.4\right)\pi i},0.5e^{\left(0.6\right)\pi i}\right),\\ \left(\left(SSW,MSS\right),0.3e^{\left(0.4\right)\pi i},0.5e^{\left(0.6\right)\pi i}\right),\left(\left(SSW,BC\right),0.3e^{\left(0.4\right)\pi i},0.5e^{\left(0.6\right)\pi i}\right),\\ \left(\left(SSW,FW\right),0.3e^{\left(0.4\right)\pi i},0.5e^{\left(0.6\right)\pi i}\right),\left(\left(SSW,SSW\right),\;0.3e^{\left(0.4\right)\pi i},0.5e^{\left(0.6\right)\pi i}\right) \end{array}\right\}$$

**Figure 9:**
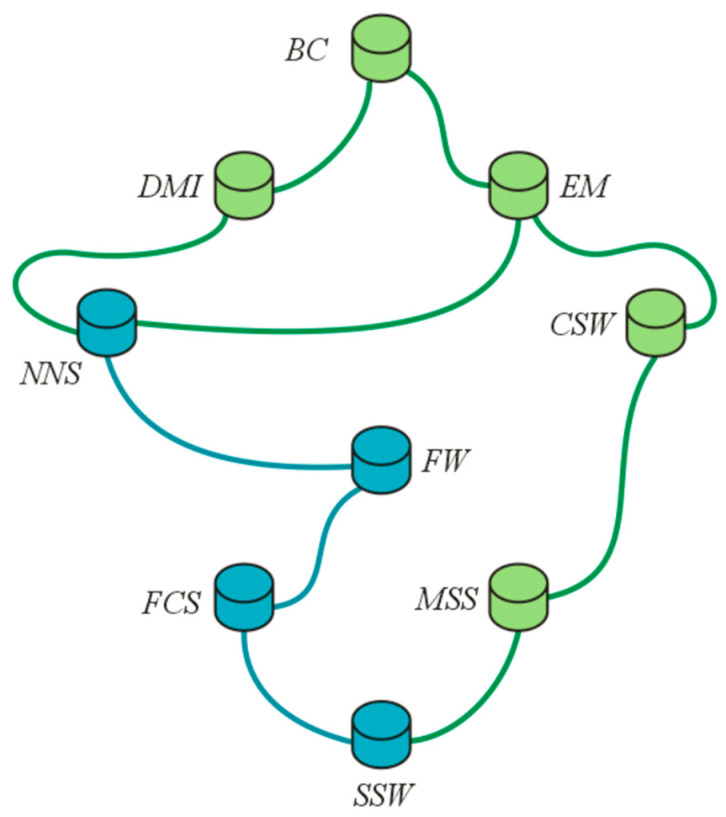
Hasse diagram for FR.

Using the Definition 13, the Hasse diagram for the above complex intuitionistic partial order FR is constructed, which is given in Figure 9. For convenience, the membership and non-membership grades are not listed in the diagram. According to R, block chain is the best security technique among the available competitors because it is the maximum as well as the maximal element (according to Definition 14), while security software such as antivirus and antimalware software provides the least security because SSW is the minimum as well as the minimal element of the diagram (according to Definition 14).

Assume that the company has certain priorities, and the following security techniques have been separated from a larger set of techniques. These security measures are listed in the subset ʝ, which is indicated in blue in the diagram.

$$ʝ=\left\{\begin{array}{c} \left(NNS,\;0.5e^{\left(0.5\right)\pi i},0.3e^{\left(0.4\right)\pi i}\right),\left(FCS,\;0.5e^{\left(0.4\right)\pi i},0.2e^{\left(0.4\right)\pi i}\right),\\ \left(FW,\;0.5e^{\left(0.6\right)\pi i},0.1e^{\left(0.2\right)\pi i}\right),\left(SSW,\;0.3e^{\left(0.4\right)\pi i},0.5e^{\left(0.6\right)\pi i}\right) \end{array}\right\}$$

The objective is to choose the best security technique among the members of set ʝ. Thus, one may be interested in the upper bounds and supremum. In this case, according to Definition 15, the upper bounds are {DMI,EM,NNS,BC}. By Definition 16, the supremum is the lowest upper bound, which is NNS. Hence, the Nozomi network solution is the most suitable cyber-security measure among the shortlisted ones that will eliminate the threats or reduce the risks of cyber-attacks.

.

## 6. Comparative Analysis

In this section, the omnipotence of the proposed framework of CIFRs is verified through a comparison of CIFRs with the existing structures such as CFRs or IFRs.

The major advantage of a CIFR over FR and IFR is the complex-valued membership and non-membership grades. The structure of a CIFR is composed of amplitude term and phase term, which enable it to model the situations with phase alteration and periodicity. On the other hand, the FRs and IFRs lack the phase term; thus, they are limited to only single dimensional models.

Meanwhile, the structure of CFRs is based on a complex number and thus consists of amplitude and phase terms. A detailed comparison between CIFRs and other structures is given in the following subsections.

### 6.1. CIFRs vs. CFRs

Let us study the relationships between the set of cyber-security techniques and cyber-crimes using the CFRs. As CFRs are superior to FRs, the comparison is carried out between CIFRs and CFRs. Consider the following two CFSs Ḯ and ʝ representing the set of security measures and the set of threats, respectively. The details of the abbreviations used in sets Ḯ and ʝ are given in Table 4 and Table 5.

$$Ḯ=\left\{\begin{array}{c} \left(DMI,\;0.7e^{\left(0.8\right)\pi i}\right),\left(EM,\;0.6e^{\left(0.6\right)\pi i}\right),\left(CSW,\;0.6e^{\left(0.4\right)\pi i}\right),\\ \left(NNS,\;0.5e^{\left(0.5\right)\pi i}\right),\left(FCS,\;0.5e^{\left(0.4\right)\pi i}\right),\left(MSS,\;0.4e^{\left(0.5\right)\pi i}\right) \end{array}\right\}$$

$$ʝ=\left\{\begin{array}{c} \left(IS,\;0.4e^{\left(0.5\right)\pi i}\right),\left(E\&DT,\;0.5e^{\left(0.6\right)\pi i}\right),\left(ECM,\;0.4e^{\left(0.4\right)\pi i}\right),\\ \left(RW,\;0.6e^{\left(0.7\right)\pi i}\right),\left(InT,0.4e^{\left(0.3\right)\pi i}\right) \end{array}\right\}$$

By applying the procedure detailed in Figure 6, the following CFR R is found, which discusses the effectiveness of security methods against each threat:


$$R=\left\{\begin{array}{c} \left(\left(DMI,IS\right),0.4e^{\left(0.5\right)\pi i}\right),\left(\left(DMI,E\&DT\right),0.5e^{\left(0.6\right)\pi i}\right),\left(\left(DMI,ECM\right),0.4e^{\left(0.4\right)\pi i}\right),\\ \left(\left(DMI,RW\right),0.6e^{\left(0.7\right)\pi i}\right),\left(\left(DMI,InT\right),0.4e^{\left(0.3\right)\pi i}\right),\left(\left(EM,IS\right),0.4e^{\left(0.5\right)\pi i}\right),\\ \left(\left(EM,E\&DT\right),0.5e^{\left(0.6\right)\pi i}\right),\left(\left(EM,ECM\right),0.4e^{\left(0.4\right)\pi i}\right),\left(\left(EM,RW\right),0.6e^{\left(0.6\right)\pi i}\right),\\ \left(\left(EM,InT\right),0.4e^{\left(0.3\right)\pi i}\right),\left(\left(CSW,IS\right),0.4e^{\left(0.4\right)\pi i}\right),\left(\left(CSW,E\&DT\right),0.5e^{\left(0.4\right)\pi i}\right),\\ \left(\left(CSW,ECM\right),\;0.4e^{\left(0.4\right)\pi i}\right),\left(\left(CSW,RW\right),\;0.6e^{\left(0.4\right)\pi i}\right),\left(\left(CSW,InT\right),0.4e^{\left(0.3\right)\pi i}\right),\\ \left(\left(NNS,IS\right),0.4e^{\left(0.5\right)\pi i}\right),\left(\left(NNS,E\&DT\right),\;0.5e^{\left(0.5\right)\pi i}\right),\left(\left(NNS,ECM\right),0.4e^{\left(0.4\right)\pi i}\right),\\ \left(\left(NNS,RW\right),\;0.5e^{\left(0.5\right)\pi i}\right),\left(\left(NNS,InT\right),0.4e^{\left(0.3\right)\pi i}\right),\left(\left(FCS,IS\right),\;0.4e^{\left(0.4\right)\pi i}\right),\\ \left(\left(FCS,E\&DT\right),0.5e^{\left(0.4\right)\pi i}\right),\left(\left(FCS,ECM\right),0.4e^{\left(0.4\right)\pi i}\right),\left(\left(FCS,RW\right),\;0.5e^{\left(0.4\right)\pi i}\right),\\ \left(\left(FCS,InT\right),0.4e^{\left(0.3\right)\pi i}\right),\left(\left(MSS,IS\right),0.4e^{\left(0.5\right)\pi i}\right),\left(\left(MSS,E\&DT\right),\;0.4e^{\left(0.5\right)\pi i}\right),\\ \left(\left(MSS,ECM\right),0.4e^{\left(0.4\right)\pi i}\right),\left(\left(MSS,RW\right),\;0.4e^{\left(0.5\right)\pi i}\right),\left(\left(MSS,InT\right),0.4e^{\left(0.3\right)\pi i}\right) \end{array}\right\}$$

Each of the elements in the above CFR demonstrates the connection between a pair of elements in an ordered pair. The effects of the first element (appearing first in the ordered pair) on the second element (appearing latter in the ordered pair) are described by the membership grades. Since the Cartesian product is carried out from the set of security measures to the set of threats, the found relation indicates the effects of the security measures on the threats. Since it is a CFR, its membership grades only display the effectiveness of a cyber-security measure against a particular threat. It also fails to provide valuable information about the ineffectiveness and failure levels of each cyber-security measure against certain threats. Meanwhile, the CIFRs provide the complete information. Hence, this shows the dominance of CIFRs over CFRs and FRs. 

### 6.2. CIFRs vs. IFRs

In this section, the IFRs and IFSs are used to investigate the matter discussed in the proposed applications, i.e., the relationships between the set of cyber-security techniques and the set of cyber-crimes. Consider the following two IFSs Ḯ and ʝ representing the set of security measures and the set of threats, respectively. The details of the abbreviations used in sets Ḯ and ʝ are given in Table 4 and Table 5.

$$Ḯ=\left\{\begin{array}{c} \left(DMI,\;0.7,0.1\right),\left(EM,\;0.6,0.3\right),\left(CSW,\;0.6,0.2\right),\\ \left(NNS,\;0.5,0.3\right),\left(FCS,\;0.5,0.2\right),\left(MSS,\;0.4,0.4\right) \end{array}\right\}$$

$$ʝ=\left\{\begin{array}{c} \left(IS,\;0.4,0.5\right),\left(E\&DT,\;0.5,0.5\right),\left(ECM,\;0.4,0.6\right),\\ \left(RW,\;0.6,0.4\right),\left(InT,0.4,0.6\right) \end{array}\right\}$$

Following the process discussed in Figure 6, the following IFR R is obtained, which discusses the usefulness of the security methods against each threat:


$$R=\left\{\begin{array}{c} \left(\left(DMI,IS\right),0.4,0.5\right),\left(\left(DMI,E\&DT\right),0.5,0.5\right),\left(\left(DMI,ECM\right),0.4,0.2\right),\\ \left(\left(DMI,RW\right),0.6,0.4\right),\left(\left(DMI,InT\right),0.4,0.6\right),\left(\left(EM,IS\right),0.4,0.5\right),\\ \left(\left(EM,E\&DT\right),0.5,0.5\right),\left(\left(EM,ECM\right),0.4,0.6\right),\left(\left(EM,RW\right),0.6,0.4\right),\\ \left(\left(EM,InT\right),0.4,0.6\right),\left(\left(CSW,IS\right),0.4,0.5\right),\left(\left(CSW,E\&DT\right),0.5,0.5\right),\\ \left(\left(CSW,ECM\right),\;0.4,0.6\right),\left(\left(CSW,RW\right),\;0.6,0.4\right),\left(\left(CSW,InT\right),0.4,0.6\right),\\ \left(\left(NNS,IS\right),0.4,0.5\right),\left(\left(NNS,E\&DT\right),\;0.5,0.5\right),\left(\left(NNS,ECM\right),0.4,0.6\right),\\ \begin{array}{c} \left(\left(NNS,RW\right),\;0.5,0.4\right),\left(\left(NNS,InT\right),0.4,0.6\right),\left(\left(FCS,IS\right),\;0.4,0.5\right), \end{array}\\ \left(\left(FCS,E\&DT\right),0.5,0.5\right),\left(\left(FCS,ECM\right),0.4,0.6\right),\left(\left(FCS,RW\right),\;0.5,\;0.4\right),\\ \left(\left(FCS,InT\right),0.4,0.6\right),\left(\left(MSS,IS\right),0.4,0.5\right),\left(\left(MSS,E\&DT\right),\;0.4,0.5\right),\\ \begin{array}{c} \left(\left(MSS,ECM\right),0.4,0.6\right),\left(\left(MSS,RW\right),\;0.4,0.4\right),\left(\left(MSS,InT\right),0.4,0.6\right) \end{array} \end{array}\right\}$$

Based on the information in the above IFR, each element shows the relationship between a pair of elements. The approach to the interpretation of the information and determination of the results is similar to the previous examples. Thus, being an IFR, it only contains the amplitude terms, and the phase terms are missing. It produces incomplete results because the duration is missing. Therefore, it only defines the effectiveness and ineffectiveness of the cyber-security measures against the threats through the membership grade and non-membership grade, respectively. This structure is unsuccessful in providing the required results. The CIFRs produce satisfactory results that are required to obtain detailed information. Hence, this example illustrates the supremacy of CIFRs over IFRs. For this reason, this article chose the structure of CIFR to analyze the matter of cyber-security and cyber-crimes in the oil and gas industries.

## 7. Conclusions

This article introduces the novel concepts of complex intuitionistic fuzzy relation (CIFR) and the Cartesian product of two complex intuitionistic fuzzy sets (CIFSs). In addition, the types of CIFRs are defined, such as equivalence, pre-order, partial order, total order, strict order relations, equivalence class and the composition of two CIFRs. Moreover, the Hasse diagram of complex intuitionistic partial order fuzzy relations is introduced. The notions of maximum, minimum, maximal, minimal, supremum and infimum, etc., are defined for a Hasse diagram. The development of these innovative frameworks and novel modeling techniques aims to address the cyber-security concerns in the oil and gas industries. These industries have recently been targeted by hackers and cyber-criminals. Thus, the current study analyzes the relationships among the effectiveness of cyber-security measures and the most serious and common risks to the mentioned industries. Then, the CIFRs are applied for the security analysis of the oil and gas industries to explore the effects of certain cyber-security measures on the threats. Moreover, the complex intuitionistic partial order fuzzy relation and the Hasse diagram are used to determine the most appropriate cyber-security method for an industry. Lastly, the proposed methods are compared with the other methods in the literature. The weaknesses of the proposed methods include the absence of a neutral grade as well as the limitations and the constraints on the sum of grades. In future, these concepts can be extended to the other generalizations of fuzzy sets [48,49,50,51], which will give rise to many interesting structures with a vast range of applications.

## Figures and Tables

**Table 1:** Summary of threats.

Threat	Abbreviation	Weakness Level	Threat Level
Infrastructure sabotage	IS	$0.4e^{\left(0.5\right)\pi i}$	$0.5e^{\left(0.5\right)\pi i}$
Espionage and data theft	E&DT	$0.5e^{\left(0.6\right)\pi i}$	$0.5e^{\left(0.2\right)\pi i}$
Ever-changing malware	ECM	$\;0.4e^{\left(0.4\right)\pi i}$	$0.6e^{\left(0.5\right)\pi i}$
Ransomware	RW	$0.6e^{\left(0.7\right)\pi i}$	$0.4e^{\left(0.2\right)\pi i}$
Insider threat	InT	$0.4e^{\left(0.3\right)\pi i}$	$0.6e^{\left(0.6\right)\pi i}$

**Table 2:** Summary of security measures.

Security Method	Abbreviation	Security Level	Risk Level
Deep Armor Industrial	DMI	$0.7e^{\left(0.8\right)\pi i}$	$0.1e^{\left(0.2\right)\pi i}$
Event Monitoring	EM	$0.6e^{\left(0.6\right)\pi i}$	$0.3e^{\left(0.3\right)\pi i}$
Cyber-security workplace	CSW	$0.6e^{\left(0.4\right)\pi i}$	$0.2e^{\left(0.4\right)\pi i}$
Nozomi Network solution	NNS	$0.5e^{\left(0.5\right)\pi i}$	$0.3e^{\left(0.4\right)\pi i}$
Forge Cyber-Security Suite	FCS	$0.5e^{\left(0.4\right)\pi i}$	$0.2e^{\left(0.4\right)\pi i}$
Managed Security Services	MSS	$0.4e^{\left(0.5\right)\pi i}$	$0.4e^{\left(0.2\right)\pi i}$

**Table 3:** Abbreviations of cyber-security measures.

Cyber-Security	Abbreviation
Deep Armor Industrial	DMI
Event Monitoring	EM
Cyber-security workplace	CSW
Nozomi Network solution	NNS
Forge Cyber-Security Suite	FCS
Managed Security Services	MSS
Block Chain	BC
Firewall	FW
Security Softwares	SSW

**Table 4:** Abbreviations of security measures in set ʝ.

Security Method	Abbreviation
Deep Armor Industrial	DMI
Event Monitoring	EM
Cyber-security workplace	CSW
Nozomi Network solution	NNS
Forge Cyber-Security Suite	FCS
Managed Security Services	MSS

**Table 5:** Abbreviations of threats in set ʝ.

Threat	Abbreviation
Infrastructure sabotage	IS
Espionage and data theft	E&DT
Ever-changing malware	ECM
Ransomware	RW
Insider threat	InT

## Data Availability

Not applicable.
